# Structural insights into inhibitory mechanism of human excitatory amino acid transporter EAAT2

**DOI:** 10.1038/s41467-022-32442-6

**Published:** 2022-08-11

**Authors:** Takafumi Kato, Tsukasa Kusakizako, Chunhuan Jin, Xinyu Zhou, Ryuichi Ohgaki, LiLi Quan, Minhui Xu, Suguru Okuda, Kan Kobayashi, Keitaro Yamashita, Tomohiro Nishizawa, Yoshikatsu Kanai, Osamu Nureki

**Affiliations:** 1 Department of Biological Science, Graduate School of Science, The University of Tokyo, Tokyo, Japan; 2 Department of Bio-system Pharmacology, Graduate School of Medicine, Osaka University, Osaka, Japan; 3 Integrated Frontier Research for Medical Science Division, Institute for Open and Transdisciplinary Research Initiative (OTRI), Osaka University, Osaka, Japan; 4 Present Address: Department of Biochemistry, The University of Oxford, Oxford, UK; 5 Present Address: Department of Molecular Pharmacology, National Institute of Neuroscience, National Center of Neurology and Psychiatry, Tokyo, Japan; 6 Present Address: Department of Applied Biological Chemistry, Graduate School of Agricultural and Life Sciences, The University of Tokyo, Tokyo, Japan; 7 Present Address: Peptidream Inc, Kawasaki, Japan; 8 Present Address: Structural Studies Division, MRC Laboratory of Molecular Biology, Cambridge, UK; 9 Present Address: Graduate School of Medical Life Science, Yokohama City University, Yokohama, Japan

**Keywords:** Cryoelectron microscopy, Membrane proteins, Permeation and transport, Transporters in the nervous system

## Abstract

Glutamate is a pivotal excitatory neurotransmitter in mammalian brains, but excessive glutamate causes numerous neural disorders. Almost all extracellular glutamate is retrieved by the glial transporter, Excitatory Amino Acid Transporter 2 (EAAT2), belonging to the SLC1A family. However, in some cancers, EAAT2 expression is enhanced and causes resistance to therapies by metabolic disturbance. Despite its crucial roles, the detailed structural information about EAAT2 has not been available. Here, we report cryo-EM structures of human EAAT2 in substrate-free and selective inhibitor WAY213613-bound states at 3.2 Å and 2.8 Å, respectively. EAAT2 forms a trimer, with each protomer consisting of transport and scaffold domains. Along with a glutamate-binding site, the transport domain possesses a cavity that could be disrupted during the transport cycle. WAY213613 occupies both the glutamate-binding site and cavity of EAAT2 to interfere with its alternating access, where the sensitivity is defined by the inner environment of the cavity. We provide the characterization of the molecular features of EAAT2 and its selective inhibition mechanism that may facilitate structure-based drug design for EAAT2.

## Abstract (web summary)

EAAT2 is an amino acid transporter implicated in glutamate homeostasis in brain and therapy resistance of cancer cells. Here, the authors report cryo-EM structures and reveal inhibitory mechanisms via selective inhibitor WAY213613.

## Introduction

Amino acids are essential biomolecules for protein biosynthesis, metabolism and signal transduction, and the control of cellular amino-acid concentrations is quite important. Human cells have eleven discrete SoLute Carrier (SLC) families that transport various kinds of amino acids across the membrane^1,2^. The SLC1A family functions as a sodium-dependent symporter for the uptake of extracellular amino acids (Supplementary Fig. 1), and its members are categorized as Excitatory Amino Acid Transporters (EAATs; EAAT1–5 function as aspartate and glutamate transporters) and Alanine Serine Cysteine Transporters (ASCTs; ASCT1 and ACST2 function as neutral amino-acid transporters)^3^.

Higher functions of the mammalian central nervous system (CNS) are linked to complex neural activities, such as learning and memory^4^. In the CNS, glutamate, a principal excitatory neurotransmitter, stimulates ionotropic receptors to elicit the postsynaptic action potential via various ion fluxes, including calcium ions^5,6^. However, excessive glutamate at synaptic clefs causes extra calcium influx. The intracellular accumulation of calcium ions is related to mitochondrial dysfunction and oxidative stress and induces neuronal cell death, known as excitotoxicity^7^. To protect neuronal cells from excitotoxity, the released glutamate is rapidly retrieved by transporters localized around the synaptic cleft. EAAT2 (also known as SLC1A2 or GLT-1) is particularly highly expressed at the plasma membrane of glial cells and removes almost all (>90%) extracellular glutamate^8–10^. Therefore, EAAT2 plays a crucial role in the extracellular glutamate homeostasis. In accordance with its essential role, a deficiency in the EAAT2 transport activity is associated with serious diseases, including psychiatric and neurological disorders^11–17^.

Structural research on the SLC1A family has revealed the architectures and transport mechanisms of the archaeal homologs (Glt_ph_ and Glt_tk_)^18–25^ and four eukaryotic transporters (thermostabilized EAAT1, EAAT3, ASCT1, and ASCT2)^26–32^. SLC1A transporters are assembled into a trimer, with each protomer consisting of scaffold and transport domains to adopt a unique alternating access model, termed the “elevator-type mechanism”^20,33^. This model is operated by the rigid elevator-like movement of the transport domain to translocate substrates across the membrane. However, despite its pivotal role in the CNS, structural information about EAAT2 has not been reported, although it is particularly needed for pharmacological studies. Recent reports demonstrated that spider venom and a novel chemical compound function as “direct activators” to increase the transport activity of EAAT2 and provide neuroprotection against excitotoxicity^34–36^. In addition to neurological diseases, although EAAT2 is mainly expressed in the brains, three tissue-specific tumors (gastric, colorectal and breast cancers) are related to the enhanced expression of EAAT2, which is associated with resistance to chemotherapeutic and endocrine therapies^37–40^. Since these resistances are clinical problems for patients, selective inhibitors of EAAT2 targeting these cancer cells might be effective drugs for cancer therapies. Furthermore, highly selective inhibitors that can discriminate EAAT2 from other EAAT transporters will be useful for basic research to elucidate the physiological importance of EAAT2. Therefore, the structures of EAAT2 will provide molecular insights to facilitate the structural-based drug design of both activators and inhibitors.

In this work, we perform cryo-EM single particle analyses to determine the structures of human EAAT2. Our structures, together with transport assays and comparisons with other EAAT structures, provide insights into the molecular features of EAAT2 and the inhibitory mechanism of the highly selective inhibitor WAY213613.

## Results and discussion

### Structural determination and overall structure

We expressed full-length wild-type human EAAT2 (HsEAAT2) with a C-terminally fused GFP tag (Supplementary Fig. 2) in Human Embryonic Kidney 293 cells (HEK293), and the recombinant proteins were purified with a GFP antibody^41^. For structural determination, purified HsEAAT2 proteins in glycol diosgenin (GDN) micelles were vitrified on grids, and 3,351 micrographs were recorded by a K3 camera. With the C3 symmetry imposed, we finally obtained the three-dimensional reconstruction map at the global resolution of 3.2 Å, based on the Fourier Shell Correlation (FSC)  =  0.143 criterion (Supplementary Fig. 3). The densities of all transmembrane (TM) helices and β-strands were clearly observed (Supplementary Fig. 4), whereas the N- and C- termini, Ala110–Ser113, Lys148–Val162, and Lys194–Val229 were not detectable, suggesting that these regions are flexible.

**Fig. 1:**
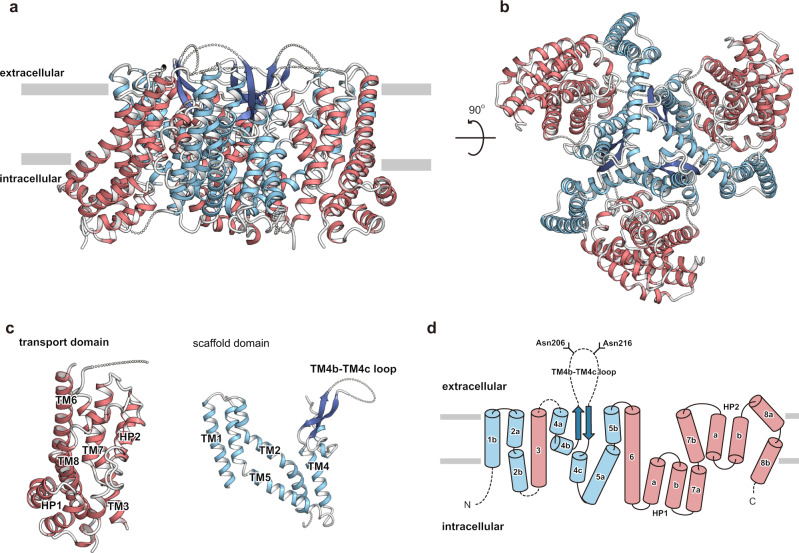
Overall structure of HsEAAT2. **a**, **b** Overall structure of HsEAAT2, as viewed from **a** the membrane plane and **b** the intracellular side. **c** The scaffold domain and the transport domain in one protomer are labeled. **d** Topology diagram of HsEAAT2. The transport domain, the scaffold domain and the β-strands of TM4 are colored light red, light blue and dark blue, respectively. Two residues (Asn206 and Asn216) in the TM4b–c are putative glycosylation sites.

HsEAAT2 forms a homotrimer (Fig. 1a, b), with each protomer consisting of eight TMs (TM1–8) and two helical hairpins (HP1 and HP2), which can be divided into transport and scaffold domains (Fig. 1c, d). The scaffold domains are located near the central symmetry axis and form the trimer interactions, while the transport domains are located at the periphery of the trimer (Fig. 1b).

**Fig. 2:**
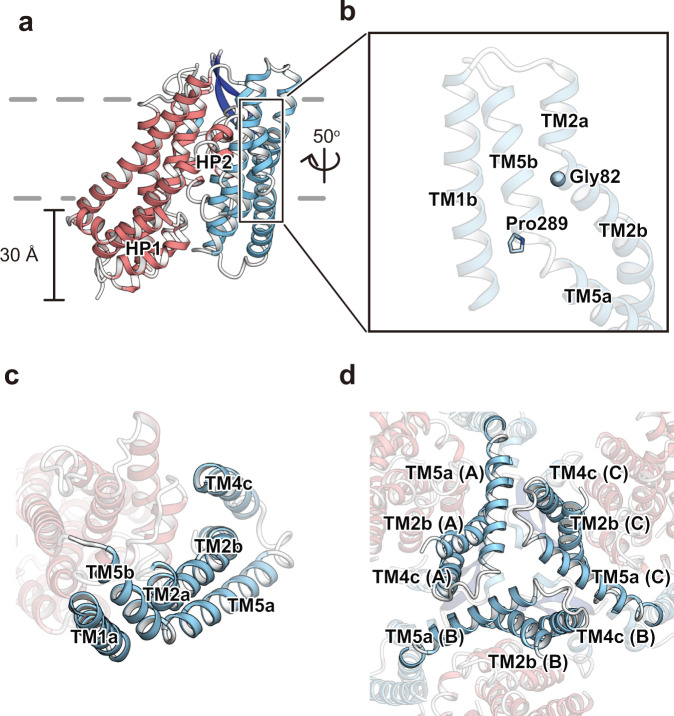
Protomer structure of HsEAAT2. **a** Overall structure of the HsEAAT2 protomer, as viewed from the membrane plane. The distance indicates the protrusion of the transport domain from the lipid membrane. **b** The “kink-inducing” residues Gly82 (TM2) and Pro289 (TM5). **c** Interactions of TM1, TM2, TM4, and TM5 in the same protomer. **d** The interactions among protomers on the intracellular side. “molA (A)–molC (C)” in brackets show each protomer.

The transport domain consists of four TMs (TM3, TM6, TM7, and TM8), HP1 and HP2 (Fig. 1c, d), which comprise HP1a, b and HP2a, b, and the connecting HP1 and HP2 loops, respectively. Two hairpins are located on the domain interface, where HP2 contacts the scaffold domain in the membrane region, while HP1 is apart from the scaffold domain and located outside of the region (Fig. 2a). The transport domains partly protrude from the lipid bilayer by about 30 Å, and the putative glutamate-binding site is open toward the intracellular solvent (Fig. 2a). Therefore, the current structure represents the inward-facing state.

The scaffold domain consists of four TMs (TM1, TM2, TM4, and TM5), and three of these TMs are divided into segments (TM2a, b, TM4a–c, and TM5a, b) (Fig. 1c, d). TM2 and TM5 are kinked by Gly82 and Pro289 (Fig. 2b) and divided into two segments, and the extracellular segments of the two helices mediate the trimeric assembly (Fig. 2b–d). In agreement with our structural information, natural variants of Gly82 and Pro289 are associated with epileptic encephalopathies^42^, suggesting that these mutations hinder the molecular trimerization.

### Substrate-free state of the transport domain

**Fig. 3:**
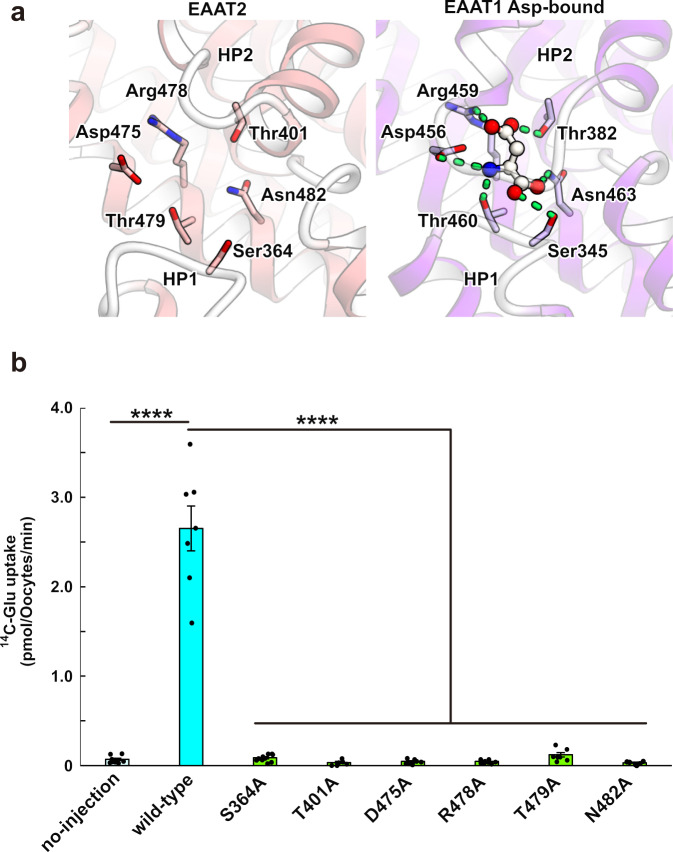
Glutamate-binding site. **a** Comparison of the substrate-binding site in HsEAAT2 with the structure of the aspartate bound-state of EAAT1 (PDB ID 5LLU). Green dots indicate interactions between aspartate and EAAT1. **b** Glutamate-uptake assay for point mutants, using *Xenopus* oocyte. Values are mean ± s.e.m. *n* =  6–10 technical replicates (*n* = 9, 7, 10, 6, 9, 8, 7, and 7 for on-injection, WT, S364A, T401A, D475A, R478A, T479A, and N482A, respectively). *p*-values are from two-sides *t* tests. 95% confidence interval. All *p*-values are ****p < 0.0001, and *p*-values of WT v.s. no-injection, S364A, T401A, D475A, R478A, T479A and N482A are 3.3 × 10^−9^, 5.9 × 10^−10^, 8.4 × 10^−7^, 3.1 × 10^−9^, 1.9 × 10^−8^, 1.8 × 10^−7^, and 1.2 × 10^−7^, respectively.

In the transport domains of SLC1A transporters, amino acid-binding sites, which recognize aspartate, glutamate and neutral amino acids, are localized in between the HP1 and HP2 loops. Recent structural studies on the inward-facing and outward-facing states of ASCT2 proposed that only the HP2 loop functions as a gate for the binding sites, and this mechanism is termed the “one-gate elevator transport mechanism”^28,29^. After the binding site closure by the HP2 loop, an elevator-like movement allows the transport of substrates across the membrane. While six highly conserved residues (Ser364, Thr401, Asp475, Arg478, Thr479 and Asn482 in HsEAAT2) constitute the putative glutamate-binding site in HsEAAT2, and the HP2 loop adopts an open conformation to allow access from the intracellular solvent, no density was observed at this site (Fig. 3a). Therefore, the current structure is likely to represent the substrate-free inward-open state. To evaluate the roles of those conserved residues, we measured the glutamate uptake activities of their point mutants, using *Xenopus* oocytes, which clearly showed that the transport activities of all mutants were essentially abolished (Fig. 3b and Supplementary Fig. 6a). These results indicate their indispensable roles in glutamate transport.

Amino acid transport by the SLC1A family members (EAATs, ASCTs and the archaeal homologs) is coupled with three sodium ions (Supplementary Fig. 1), and their binding sites have been clearly identified in previous structural studies^19,20,23,26,30^. In addition to sodium ions, EAATs utilize an extracellular H^+^ gradient, and its coupling mechanism was clarified in a recent report on EAAT3^30^. Firstly, in the occluded state, the HP2 loop functions as the gate to allow the binding of the transported amino acid to the site. Next, the HP2 loop adopts the open conformation to release the amino acid substrate, termed “IFS-Na^+^” in the previous work. In this state, the coupling of H^+^ neutralizes the charged glutamate residue (Glu405 and Glu374 in HsEAAT2 and EAAT3, respectively), whose protonation prevents the formation of a salt bridge with an arginine residue (Arg478 and Arg447 in HsEAAT2 and EAAT3, respectively) involved in the amino-acid substrate recognition. Upon the H^+^ release, the arginine residue adopts a different conformation to form the salt bridge with the deprotonated glutamate residue.

In our HsEAAT2 structure, the local structures of the three residues (Met398, Glu405 and Arg478) are similar to those in IFS-Na^+^ of EAAT3 (Supplementary Fig. 7). The p*K*_*a*_ value of Glu405 calculated by the PROPKA program^43^ is 7.0, which is the same pH value in our purification. Considering the structural information and the p*K*_*a*_ value, Glu405 is probably in a transition between deprotonated and protonated forms, and does not stably form the salt bridge with Arg478. Therefore, our HsEAAT2 structure resembles the IFS-Na^+^ state of EAAT3. Since the transport domain adopts almost the same conformations in both the inward- and outward-facing states, behaving as a rigid body during the transport cycle^20,33^, a similar arrangement of the substrate-binding site may be observed in the outward-facing state of HsEAAT2.

### Putative functions of TM4 loop and lipid-binding sites

All eukaryotic SLC1A transporters have a long loop between the TM4b and TM4c segments (TM4b–4c loop; Cys184–Asn241 in HsEAAT2), whereas such loops are not conserved in the archaeal homologs, Glt_ph_ and Glt_tk_ (Supplementary Fig. 2). In HsEAAT2, only the juxtamembrane portion of this loop forms antiparallel β-strands (Gln186–Lys193 and Lys231–Asp238) protruding from the scaffold domain, and the rest of the loop, including two potential glycosylation sites (Asn206 and Asn216), is completely disordered (Fig. 1a, c, d). The N206S mutation and a reduced glycosylation phenotype have been detected in neurological disorders^44,45^. Consistently, the N206S mutation hampers localization in the plasma membrane, hence causing a marked reduction in the EAAT2-mediated glutamate uptake^46^. Therefore, the β hairpin may structurally support the association of the flexible glycosylated loop with luminal and/or extracellular proteins during proper anterograde transport and the endocytic events involving recycling to the plasma membrane, respectively.

The localizations and activities of transporters are affected by specific lipid environments, such as lipid composition and membrane thickness^47^. Some structural studies of SLC1A transporters reported lipid-binding sites^26–28^, and similarly, we observed two flat-shaped densities within each protomer. The density shapes and sizes suggested that they are likely derived from GDN and endogenous cholesterol (Supplementary Fig. 5). GDN is located between the transport and scaffold domains, and the cholesterol is on the cytoplasmic end of the scaffold domain, where it forms an interaction with Trp286 (TM5) (Supplementary Fig. 5b, c). Similar cholesterol-binding sites were observed in ASCT2 and EAAT1 (refs. 27, 28, 32,). Trp272 (the corresponding residue of EAAT2 is Met283, which is 1-turn apart from Trp286 of EAAT2) and Trp287 are located on the TM5s of ASCT2 and EAAT1, respectively, and these residues form similar interactions with the cholesterol molecules. The tryptophan residues located at the corresponding position of TM5 are highly conserved among eukaryotic SLC1A transporters (Supplementary Fig. 2), suggesting their importance among the family members. To assess its role, we mutated Trp286 to alanine and investigated the transport activity, using *Xenopus* oocytes. While both the expression level and localization of W286A were similar to those of the wild type, the glutamate uptake by the mutant was reduced (Supplementary Fig. 5e–g). This result suggests that the mutation of Trp286 has negative effects on the transport activity in plasma membrane environments. EAAT2 tends to localize at cholesterol-rich microdomains, where cholesterol molecules are essential to sustain the transport activity^48,49^. Consistently, the reduction of EAAT2 activity in cholesterol-depleted membranes was reportedly observed in people with Alzheimer’s disease^50^. Since GDN has a cholesterol-like moiety, we hypothesize that the GDN- and cholesterol-binding sites identified in the current structure could function as the binding sites for endogenous cholesterol, and probably contribute to the localization and/or structural stability and the transport activity of EAAT2. In particular, the intracellular side of TM5 commonly participates in the cholesterol binding.

### Inward-facing WAY213613-bound (IFS-WAY213613) state

**Fig. 4:**
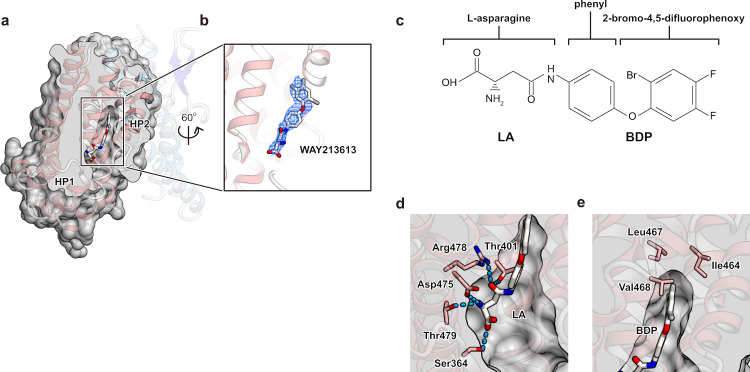
IFS-WAY213613 state. **a**, **b** Structure of the IFS-WAY213613 state. **a** Cut-away representation of the cavity with WAY213613. **b** Close-up views of the WAY213613-bound site. The *F*_*o*_ – *F*_*c*_ omit map of WAY213613 is contoured at 3.0 σ (normalized within mask), and shown as a blue mesh. **c** The structure of WAY213613. WAY213613 is composed of the l-asparagine (LA) moiety and the (2-Bromo-4,5-difluorophenoxy) phenyl (BDP) moiety. **d**, **e** Recognition of the LA site of WAY213613. **d** Substrate-binding site recognizing the LA moiety of WAY213613. **e** Close-up view of the BDP moiety of WAY213613.

The transport activities of EAATs are blocked by various inhibitors. For instance, TFB-TBOA is one of the strongest inhibitors for EAATs; it significantly suppresses the activities of not only EAAT2 but also other EAATs (IC_50_ values are 17, 22, and 300 nM for EAAT2, EAAT1, and EAAT3, respectively)^51^. Recently, WAY213613 was developed as a highly selective inhibitor of EAAT2 (IC_50_ values are 85, 5004, and 3787 nM for EAAT2, EAAT1, and EAAT3, respectively, in the HEK cell line)^52^. Among the available inhibitors, WAY213613 is the most potent and selective for EAAT2. However, its selective inhibitory mechanism for EAAT2 is unclear. To clarify the underlying inhibitory mechanism, we determined the cryo-EM structure of HsEAAT2 complexed with WAY213613 at 2.8 Å (Supplementary Fig. 8). The root mean square deviation value with the substrate-free state is 0.399 Å, indicating that the structures are very similar. Consistently, the transport domains represent the inward-facing states bound with WAY213613 (IFS-WAY213613 state), and both the *F*_*o*_ – *F*_*c*_ omit map calculated by the Servalcat program^53^ and the raw map show that WAY213613 is located in between HP1 and HP2 (Fig. 4a, b and Supplementary Fig. 8d). WAY213613 is composed of two moieties: L-asparagine (LA) and 4-(2-bromo-4,5-difluorophenoxy) phenyl (BDP) moieties (Fig. 4c). The LA moiety is recognized by five residues (Ser364, Thr401, Asp475, Arg478 and Thr479) at the glutamate-binding site (Fig. 4a, d), which are completely conserved among EAAT1–3 (Supplementary Fig. 2) and important for the glutamate transport (Fig. 3b). On the other hand, the BDP moiety is accommodated in a cavity located near the glutamate-binding site (Fig. 4a, e), formed by the end of HP2b, TM7 and TM8.

**Fig. 5:**
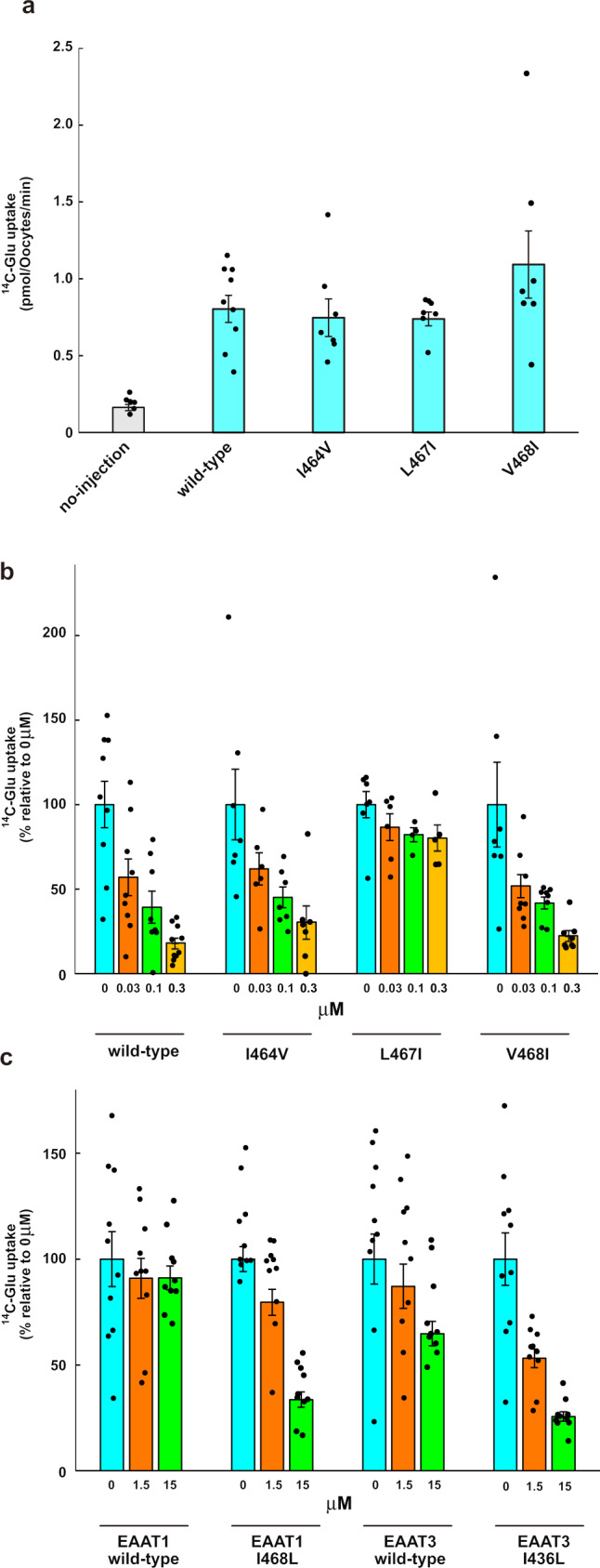
Sensitivity of WAY213613. **a** Glutamate-uptake of each mutant, using *Xenopus* oocytes. Values are mean ± s.e.m. *n* =   6–9 technical replicates (*n* = 6, 9, 7, 7, and 7 for no-injection, WT, I463V, L467, and V468I). **b** Glutamate-uptake assay for point mutants treated with WAY213613. Values of vertical and horizontal axes are relative to each 0 μM and concentration points (0, 0.03, 0.1, and 0.3 μM) of WAY213613, respectively. Values are mean ± s.e.m. *n* =  4–10 technical replicates (*n* = 9, 9, 8, and 10 for WT in 0, 0.03, 0.1, and 0.3 μM, respectively. *n* = 7, 6, 7, and 7 for I463V in 0, 0.03, 0.1, and 0.3 μM, respectively. *n* = 7, 6, 4, and 5 for L467I in 0, 0.03, 0.1, and 0.3 μM, respectively. *n* = 6, 9, 8, and 8 for V468I in 0, 0.03, 0.1, and 0.3 μM, respectively). **c** Glutamate-uptake assay for point mutants of EAAT1 and EAAT3 treated with WAY213613. Values of vertical and horizontal axes are relative to each 0 μM and concentration points (0, 1.5, and 15 μM) of WAY213613, respectively. Values are mean ± s.e.m. *n* =  10 technical replicates for each transport activity.

While the residues located around the BDP moiety are similarly conserved in EAATs (Supplementary Figs. 2 and 9), we found slight variations in three residues (Ile464, Leu467 and Val468), which are substituted with different sets of residues in EAAT1 and EAAT3 (Supplementary Fig. 2). As these residues are close to the tip of the 2-bromo-4,5-difluorophenoxy group of the BDP moiety (Fig. 4c, e), we supposed that the selectivity among EAAT1–3 may depend on the BDP moiety rather than the LA moiety. To verify our hypothesis, we designed point mutants, in which these residues are substituted with the corresponding residues of EAAT1 and/or EAAT3, and investigated the inhibitory effects of WAY213613 on the mutants, using *Xenopus* oocytes. All mutants showed similar transport activities to the wild type in the absence of WAY213613 (Fig. 5a), experimentally confirming that the mutations at the cavity have no or minimal effects on the glutamate uptake. The inhibition by WAY213613 was more or less affected by all three mutants (I464V, L467I and V468I). For instance, in the L467I mutant, although the IC_50_ value for EAAT2 is 130 nM in *Xenopus* oocytes^52^, the transport activity is hardly inhibited by WAY213613, showing almost the same level of glutamate uptake as the control even with the highest concentration (300 nM) of WAY213613 (Fig. 5b and Supplementary Fig. 6b). According to the sequence alignment, the residue corresponding to Leu467 in EAAT2 is substituted with isoleucine in EAAT1, EAAT3 and all other SLC1A transporters. Since the L467I mutation of EAAT2 decreased the WAY213613 sensitivity, the “EAAT2-like” mutations (isoleucine to leucine) of EAAT1 and EAAT3 may change the sensitivities to WAY213613. To assess this possibility, we generated “EAAT2-like” mutants of EAAT1 (I468L) and EAAT3 (I436L) and measured their glutamate uptake in the presence of WAY213613, using oocytes. While the uptake activities of wild type EAAT1 at 1.5 and 15 μM were not significantly affected, those of the I468L mutant were decreased in a concentration-dependent manner (Fig. 5c and Supplementary Fig. 6c). In addition, whereas the wild type EAAT3 showed only a slight decrease in the uptake activities by WAY213613, the decrease became much more prominent in the I436L mutant. Therefore, these leucine mutations in EAAT1 and EAAT3 remarkably change the WAY213613 sensitivities. These results suggest that the residues forming the cavities are closely related to the sensitivities of the EAAT subtypes to WAY213613.

### Inhibitory mechanism of WAY213613

**Fig. 6:**
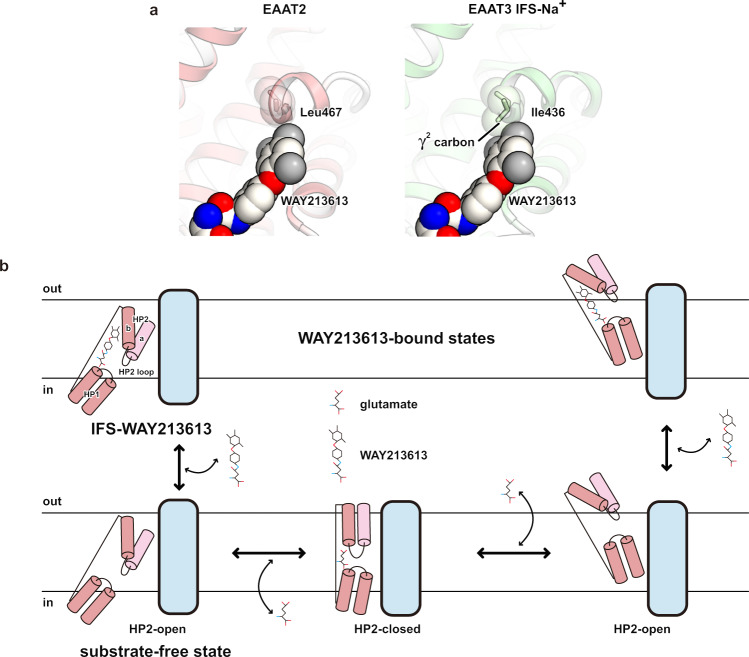
Inhibition mechanism by WAY213613. **a** Close-up view of the IFS-WAY213613 state and molecular superposition of EAAT3 IFS-Na^+^. WAY213613, Leu467, (HsEAAT2) and Ile436 (EAAT3) are shown as CPK models. **b** In the HP2-open configuration of the outward-facing state, extracellular glutamate is accessible at the substrate-binding site, and subsequently, HP2 is closed to transport glutamate into the intracellular solvent (bottom model). WAY213613 inhibits the movement of the HP2 loop (top model).

The LA moiety occupies the glutamate-binding site, where it probably competes with the glutamate binding, while the BDP moiety occupies the cavity near this binding site (Fig. 4d, e). A similar cavity is also present in IFS-Na^+^ of EAAT3 (Supplementary Fig. 10a, b). The mutation analysis demonstrated that the inhibitory effect of WAY213613 is largely diminished when Leu467 is substituted with the corresponding residues of EAAT1 and EAAT3 (isoleucine) (Fig. 5a, b and Supplementary Fig. 2). Considering the molecular superposition between the IFS-Na^+^ state of EAAT3 and the IFS-WAY213613 state of EAAT2, the γ^2^ carbon of isoleucine would narrow the cavity and sterically interfere with the binding of the 2-bromo-4,5-difluorophenoxy group of the BDP moiety (Fig. 6a). Consistently, mutants (isoleucine to leucine) of EAAT1 and EAAT3 are sensitive to WAY213613 (Fig. 5c). Altogether, the selectivity of WAY213613 is determined by the different local environments within the cavities among EAATs. In EAAT1 and EAAT3, slight changes would prevent the proper accommodation of the BDP moiety and thus lead to the lower affinity for WAY213613.

The conformation of the HP2 loop in the IFS-WAY213613 state is very similar to that in the substrate-free state. The comparison among this IFS-WAY213613 state of EAAT2 and the Asp-bound states of EAAT1 and EAAT3 revealed that the end of HP2b slightly moves towards TM7 and TM8 upon aspartate binding, which accompanies the closing of the HP2 loop gate (Supplementary Fig. 10b, c). The amino acid moieties of other competitive inhibitors, TBOA and TFB-TBOA, are also captured by the glutamate-binding sites of the transport domains^19,26^. In the TFB-TBOA-bound state of EAAT1, the non-amino acid moiety of TFB-TBOA inhibits the movement of the HP2 loop. However, the arrangement of HP2b could not accommodate the BDP moiety (Supplementary Fig. 10d). While WAY213613 is the selective inhibitor for EAAT2, TBOA and TFB-TBOA broadly interfere with SLC1A transporters^54,55^. Considering our results from EAAT1–3, the selective effects on WAY213613 are related to the environments of their cavities (Fig. 5b, c), suggesting that the inhibitory mechanisms of WAY213613 are different from those of other non-selective inhibitors, such as TBOA and TFB-TBOA. When the BDP moiety is captured by the cavities, the moiety is likely to sterically interfere with the movements of the HP2b helix, indicating that WAY213613 indirectly prevents the HP2 loop gating of EAATs. In summary, the LA and BDP moieties of WAY213613 play distinct roles in the EAAT2 inhibition, by competing with the glutamate binding and sterically preventing the HP2 loop gating, respectively. Closure of the HP2 loop is essential for the elevator-like movement of the transport domain^29^, suggesting that the BDP moiety locks the conformation of the HP2 loop to suspend the transport cycle. Whereas our structure complexed with WAY213613 represents the inward-facing state, the presence of WAY213613 in the extracellular solution clearly affected both the wild type and mutants in the oocyte assay (Fig. 5b). As the transport domains of both the inward- and outward-facing states adopt almost the same conformations and hence behave as rigid bodies during the transport cycle^20^, WAY213613 could bind the transport domain in the outward-facing state (Fig. 6b).

### Characterization of molecular features of EAAT2

Despite the essential role of glutamate as a neural transmitter in the CNS, excessive glutamate is toxic to neurons. EAAT2 clears almost all extracellular glutamate to maintain a low concentration, and dysfunction of the transporter leads to numerous neurological disorders. Therefore, extensive research has been conducted to understand the mechanism of EAAT2. In this work, we determined the structures of EAAT2 in the substrate-free and the inhibitor-bound states, and clarified the structural basis for its molecular features. Especially, the WAY213613-bound structure revealed the characteristic inhibitory mechanisms by its two moieties.

We observed the densities of cholesterol-related molecules in the present HsEAAT2 structure. Especially, Trp286, which is highly conserved among EAATs, interacts with cholesterol molecules and is related to the transport activity (Supplementary Fig. 5). A large portion of EAAT2 in the plasma membrane prefers to be localized at lipid rafts, reportedly affecting glutamate transport^48^. The detailed mechanism explaining why EAAT2 requires cholesterol molecules is still unclear. However, a recent report found that cholesterol molecules enhance the transport rate of ASCT2, showing that these molecules facilitate the elevator-like movement of the transport domains^56^. Since the conformational change of the transport domain induces the local membrane deformation^24^, the cholesterol may change the transport dynamics by altering the membrane properties^57^, to facilitate the uptake of extracellular glutamate. Therefore, our structural information will help to clarify the relationships between EAAT2 and lipid molecules that enhance the proper localization and activity of EAAT2 and provide insights into lipid interactions with eukaryotic SLC1A transporters.

According to the inhibitory mechanism of WAY213613, more selective inhibitors for EAAT2 or other EAAT subtypes could be developed by modifying the moiety corresponding to BDP in WAY213613. Indeed, the different residues around the tip of the BDP are related to the WAY213613 sensitivities (Fig. 5), and these results could support strategies for BDP modifications. WAY213613 has not yet been tested in animal experiments to evaluate its potential to permeate the blood brain barrier (BBB) and cause excitotoxicity. The BBB permeability of EAAT2 inhibitors would have the potential risk of excitotoxicity. However, WAY213613 is predicted to not pass through the BBB, based on the SwissADME server, which includes the BOILED-Egg prediction program judging BBB permeability based on the lipophilicity and polarity of small molecules^58,59^. The relationship between cancers and glutamate is gradually being elucidated, highlighting the possibility of anti-cancer drug development using glutamate-targeted but BBB-impermeable compounds^60^. Recent studies have revealed that EAAT2 is upregulated in at least three types of tumors (gastric, colorectal and breast cancers)^37–40^, and other cancers might utilize EAAT2 and/or other EAATs for their aggressive phenotypes including, but not limited to, malignant progression and resistance to chemo- and endocrine therapies. Consistently, research published in 2022 reported that EAAT3 enhances tumor angiogenesis^61^. Therefore, although further experiments to reveal the BBB impermeability and anti-cancer effects are required, WAY213613 and its derivative compounds may pave the way for future research and/or strategies for cancer therapies. Furthermore, since the recent work suggested that EAAT2 is potentially expressed in retinal and peripheral tissues (liver, lung, and testis)^62^, such selective inhibitors will reveal more details of the physiological roles of EAAT2 in the extracellular glutamate homeostasis of not only the brain but also other organs.

## Methods

### Purification of HsEAAT2

The sequence encoding the full-length human EAAT2 isoform 1 (SLC1A2; Uniprot ID P43004) was amplified from a human brain complementary DNA library (Zyagen) and inserted into the pEG BacMam vector^63^, with the C-terminally fused tobacco etch virus (TEV) protease cleavage site, enhanced green fluorescent protein (eGFP) and His_8_-tag. Baculoviruses were generated in *Spodoptera frugiperda* Sf9 cells, using the Bac-to-Bac system (Invitrogen).

HEK293 GnTI^-^ cells were grown and maintained in FreeStyle 293 medium (Gibco) supplemented with 2% fetal bovine serum, with 8% CO_2_ under humidified conditions. P2 baculoviruses were added to cells at a density of ~3.0 × 10^6^ cells/mL. Cells were cultured at 37 ^o^C for 24 h. To boost overexpression, sodium butyrate was added at a final concentration of 10 mM, and cells were cultured at 30 ^o^C for 48 h. Cells were collected by centrifugation at 5000 × *g* for 6 min, resuspended in Buffer A (50 mM HEPES-NaOH, pH 7.0, 300 mM NaCl, and 10% glycerol) and disrupted by probe sonication for 3 min. The debris was removed by centrifugation (8000 × *g*, 10 min, 4^ o^C), and the membrane was subsequently collected by ultracentrifugation (125,000 × *g*, 1 h, 4 ^o^C). The membrane pellet was resuspended in Buffer A, homogenized in a glass homogenizer, and stored at −80^ o^C.

All purification procedures were performed at 4 ^o^C. The membrane fraction was solubilized for 1 h in Buffer A containing 1.0% lauryl maltose neopentyl glycol. After the insoluble material was removed by ultracentrifugation (125,000 × *g*, 30 min, 4^ o^C), the supernatant was incubated with CNBr-Activated Sepharose 4 Fast Flow beads (GE Healthcare) coupled with an anti-GFP nanobody^41^ for 3 h. The resin was poured into an open column and washed with 15 column volumes of Buffer A containing 0.2% glycol diosgenin (GDN). The resin was mixed overnight with both TEV protease and Buffer A containing 2.0 mM dithiothreitol (DTT) and 0.2% GDN. The resin was poured into an open column, and the eluate was collected and concentrated for subsequent gel filtration chromatography (Superose 6 Increase 10/300 GL, GE Healthcare) in SEC Buffer (50 mM HEPES-NaOH, pH 7.0, 300 mM NaCl, 2.0 mM DTT and 0.05% GDN). The fraction containing the HsEAAT2 proteins was pooled and concentrated to 5–7 mg mL^−1^ using an Amicon Ultra Filter (MWCO 100 kDa). In the structural analysis of IFS-WAY213613, the HsEAAT2 protein was incubated with 1.0 mM WAY213613 (Tocris) for 1 h on ice.

### Sample vitrification and cryo-EM data acquisition

The purified HsEAAT2 proteins were applied onto freshly glow-discharged Quantifoil holey carbon grids (R1.2/1.3, Cu/Rh, 300 mesh), which were blotted for 4 s at 4 °C in 100% humidity and plunge-frozen in liquid ethane by using Vitrobot Mark IV (Thermo Fisher Scientific).

The grids were transferred to a Titan Krios G3i microscope (Thermo Fisher Scientific), running at 300 kV and equipped with a Gatan BioQuantum Energy Filter (GIF) and a Gatan K3 direct electron detector in the electron counting mode. Imaging was obtained at a nominal magnification of 105,000×, corresponding to a calibrated pixel size of 0.83 Å/pix. Each image was dose-fractionated to 63 (substrate-free state) or 48 (IFS-WAY213613) frames at a dose rate of 15 e^−^ per pixel per second, to accumulate a total dose of ~50 e^−^ Å^−2^. The data were automatically acquired by the image shift method using the SerialEM software, with a defocus range of −0.8 to −1.6 μm.

### Data processing and model building

Image processing was performed in RELION-3.1 (ref. 64). The movie frames were aligned in 4 × 4 patches and dose-weighted with RELION’s implementation of the MotionCor2 algorithm^65^, and defocus parameters were estimated by CTFFIND-4.1.13 (ref. 66). For the substrate-free state, template-based auto-picking was performed with the 2D class averages of a few hundred manually picked particles as templates. A total of 1,070,865 particles were extracted in 3.1125 Å pix^−1^. These particles were subjected to one round of 2D classification. The initial 3D reference map was generated in RELION. Subsequently, selected 374,675 particles were re-extracted in the pixel size of 1.55625 Å pix^−1^ and further subjected to 3D classification with C1 symmetry. Then, 135,571 particles were refined with C3 symmetry. The resulting 3D models and particle sets were subjected to Bayesian polishing^67^, CTF refinement (refinements of magnification anisotropy, optical aberration and per-particle defocus), and 3D refinement. After micelle subtraction and 3D classification without alignment (C1 symmetry), 91,390 particles were selected. CTF refinement and 3D refinement with SIDESPLITTER^68^ yielded a map with a global resolution of 3.2 Å, with the gold standard Fourier shell correlation criteria (FSC  =  0.143). For the WAY213613-bound state, a total of 831,890 particles were initially extracted in 3.1125 Å pix^−1^, and subjected to 2D classification and 3D classification with C3 symmetry. The following processes were the same as those used for the substrate-free state dataset. The selected 527,996 particles were subjected to 3D refinement, Bayesian polishing and CTF refinement. After micelle subtraction and 3D classification without alignment (C1 symmetry), 84,340 particles were selected. Finally, iterative CTF refinement and 3D refinement with SIDESPLITTER yielded a 2.8 Å resolution map. The initial structural model of HsEAAT2 was built by the Phyre2 program^69^, based on human ASCT2 (PDB 6GCT). After model fitting by the MOLREP program^70^, the models were manually readjusted using COOT^71^, and then refined using PHENIX^72^. The model and restraint information of WAY213613 were generated by the eLBOW program^73^. Finally, the models and the *F*_*o*_ – *F*_*c*_ omit map of WAY213613 were refined and generated, respectively, by Refmac5 (ref. 74) using Servalcat^53^ under C3 symmetry constraints. The figures depicting the molecular structures were prepared using Chimera or CueMol (http://www.cuemol.org/).

### Transport measurements and expression analyses in *X. laevis* oocytes

The EAAT2 coding sequence was amplified by PCR from pEG BacMam-HsEAAT2 with the following primer pair: forward 5’- GGGGGATCC*GCCACC*ATGGCATCTACGGAAGGTGCCAAC-3’ (BamHI site underlined, Kozak sequence in italics) and reverse 5’-CCCGAATTCTCATTTCTCACGTTTCCAAGGTTCTTC-3’ (EcoRI site underlined). The PCR product was cloned into pcDNA3.1(+) (Invitrogen) at BamHI/EcoRI sites to obtain pcDNA3.1(+)-HsEAAT2. Synthesized EAAT1 and EAAT3 genes were inserted into pcDNA3.1(+), with 5’-GGATCC*GCCACC* (BamHI site underlined, Kozak sequence in italics) and 3’-GGCAGCAGCGGCTACCCATACGATGTTCCAGATTACGCT*TGA*GAATTC (GSSG linker and HA tag, stop codon in italics, EcoRI site underlined) added. Mutations were introduced by whole-plasmid PCR using PrimeSTAR MAX DNA polymerase (Takara) according to the manufacturer’s protocol. Amino acid substitutions to alanine (for W286A, S364A, T401A, D475A, R478A, T479A and N482A in EAAT2), valine (for I464V in EAAT2), isoleucine (for L467I and V468I in EAAT2) and leucine (I468L and I436L in EAAT1 and EAAT3, respectively) were performed by altering the corresponding codons into GCA (GCG for W286A), GTA, ATC and CTG, respectively.

Transport measurements and expression analyses in *X. laevis* oocytes were performed as described previously with minor alterations^75^. cRNAs of EAAT1, EAAT2 and EAAT3 (wild-type and mutants) were synthesized in vitro from EcoRI-linearized plasmids, polyadenylated, and injected into defolliculated oocytes (25 ng/oocyte). The uptake measurements were performed 3d after injection, in ND96 buffer (96 mM NaCl, 2 mM KCl, 1.8 mM CaCl_2_, 1 mM MgCl_2_, 5 mM HEPES, pH 7.4) containing 50 μM of ^14^C-Glutamate (24.2 Ci mol-1, ARC, St. Louis, U.S.A.) for 30 min at room temperature. For inhibition experiments, the uptake of 10 μM of ^14^C-Glutamate was measured with or without the indicated concentrations of WAY213613 (Tocris, Bio-Techne, US). Protein expression from the injected cRNAs was analyzed by immunoblotting in total membranes. For EAAT2, an anti-EAAT2 antibody (sc-365634, 1:400, Santa Cruz Biotechnology) and peroxidase goat anti-mouse IgG (AB_10015289, 1:10,000, Jackson ImmunoResearch) were used. For EAAT1 and EAAT3, an anti-HA antibody (#561, 1:1,000, MBL Life Science) and peroxidase goat anti-rabbit IgG (AB_2307391, 1:10,000, Jackson ImmunoResearch) were used. Localizations of the expressed proteins were analyzed by immunofluorescence on paraffin sections. Antigen retrieval was performed with citrate buffer (0.01 M, pH 6.0) at 121 °C for 5 min. For EAAT2, an anti-EAAT2 antibody (sc-365634, 1:200, Santa Cruz Biotechnology) and Alexa Fluor 568-conjugated anti-mouse IgG (A11031, 1:2000, Invitrogen) were used. For EAAT1 and EAAT3, an anti-HA antibody (#561, 1:200, MBL Life Science) and Alexa Fluor 488-conjugated anti-rabbit IgG (A21206, 1:2000, Invitrogen) were used. Images were acquired using a fluorescence microscope (BZ-9000, Keyence) equipped with a ×40 objective lens (CFI Plan Apo λ, numerical aperture 0.95, Nikon).

### Reporting summary

Further information on research design is available in the Nature Research Reporting Summary linked to this article.

## Supplementary information

Supplementary Information

Peer Review File

Reporting Summary

## Data Availability

The cryo-EM density maps and atomic coordinates have been deposited in the Electron Microscopy Data Bank (EMDB). The accession codes for the maps are EMD-32098 (the substrate-free state) and EMD-32097 (the IFS-WAY213613 state). The PDB accession codes for the coordinates are 7VR8 (the substrate-free state) and 7VR7 (the IFS-WAY213613 state). The raw images have been deposited in the Electron Microscopy Public Image Archive, under accession code EMPIAR-11084. Source data are provided with this paper.

## Source data

Source Data
