# Supplementary material for: Structural insights into inhibitory mechanism of human excitatory amino acid transporter EAAT2
Source: Nat Commun. 2022 Aug 11;13:4714. doi: 10.1038/s41467-022-32442-6 (PMC9372063; doi:10.1038/s41467-022-32442-6)

Supplementary information

Structural insights into inhibitory mechanism of human excitatory amino acid transporter EAAT2

Kato et al.,

Supplementary Figures 1-10

Supplementary Table 1

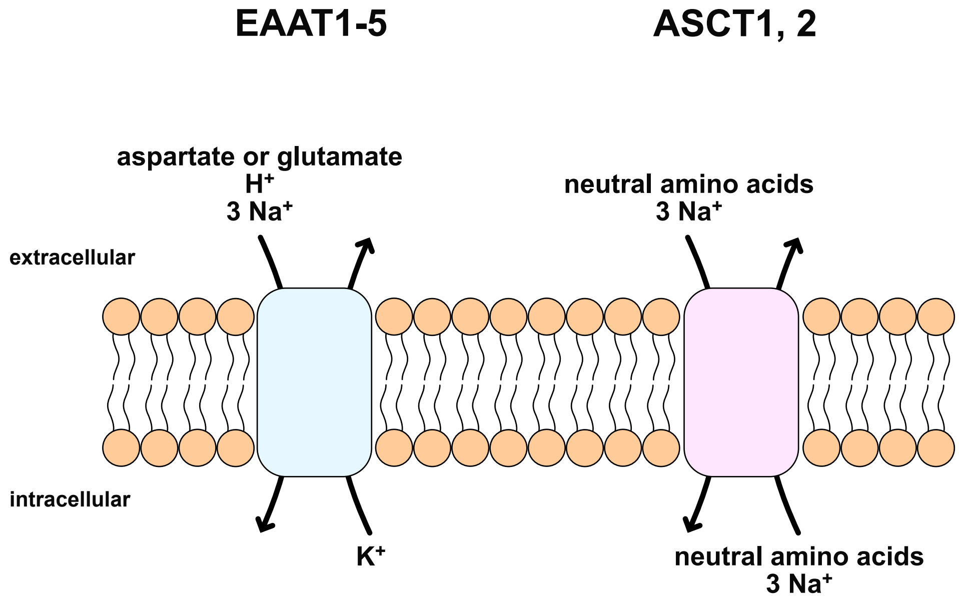

Supplementary Figure 1 │ Substrates and coupling ions of SLC1A transporters

Amino acid transport by all SLC1As is coupled to three extracellular sodium ions. In addition to sodium ions, extracellular proton and intracellular potassium ions are utilized for the transport cycle of EAATs.

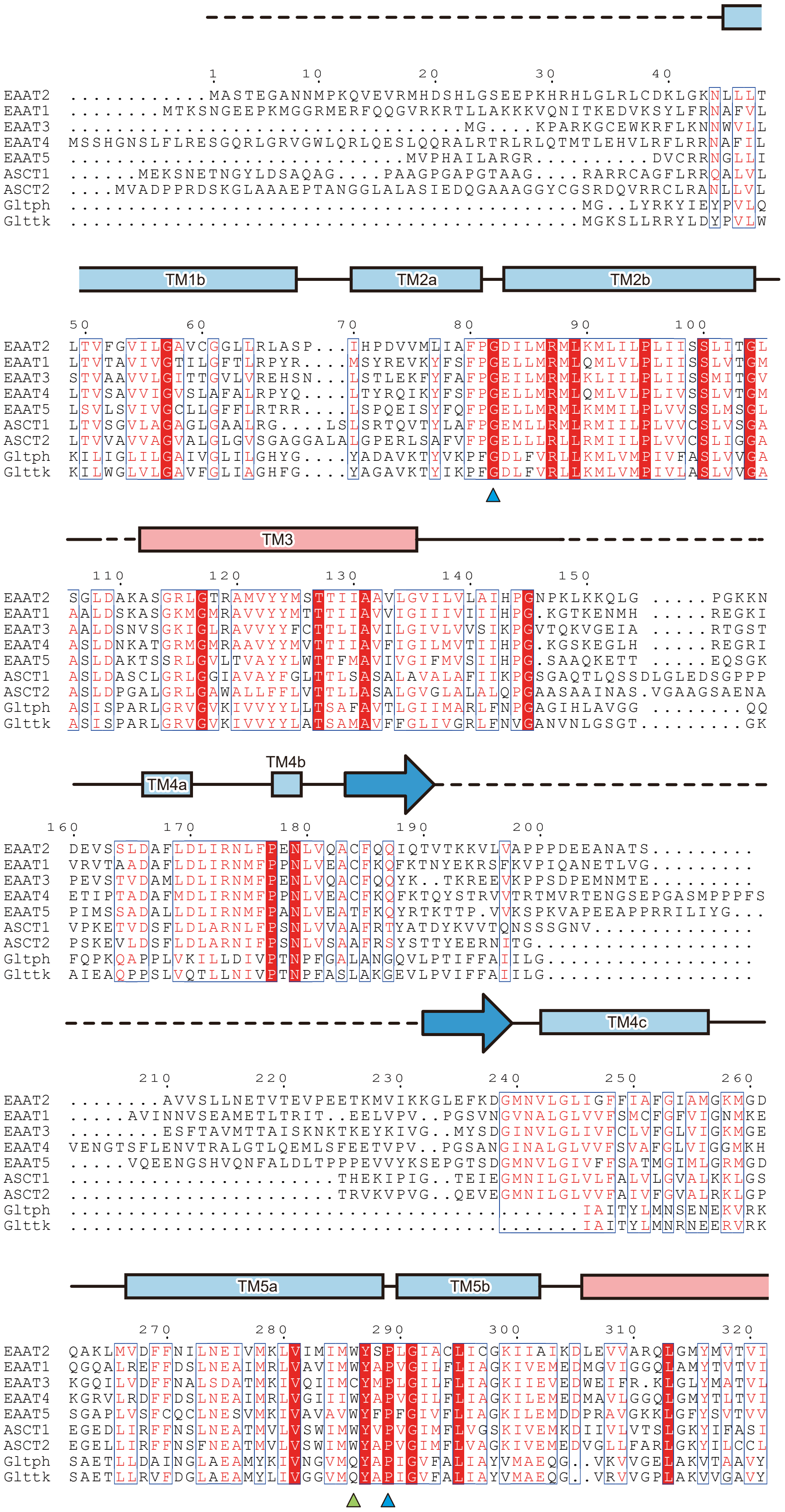

1 10 20 30 40
EAAT2 ............MASTEGANNMPKQVEVRMHDSHLGSEEPKHRHLGLRLCDKLGKNLLLT
EAAT1 ........MTKSNGEEPKMGGRMERFQQGVRKRTLLAKKKVQNITKEDVKSYLFRNAFVL
EAAT3 ....MG....KPARKGCEWKRFLKNNWVLL
EAAT4 MSSHGNSLFLRESGQRLGRVGWLQRLQESLQQRALRTRLRLQTMTLEHVLRFLRRNAFIL
EAAT5 ....MVPHAILARGR.........DVCRRNGLLI
ASCT1 ......MEKSNETNGYLDSAQAG....PAAGPGAPGTAAG....RARRCAGFLRRQALVL
ASCT2 ....MVADPPRDSKGLAAAEPTANGGLALASIEDQGAAAGGYCGSRDQVRRCLRANLLVL
Gltph ....MG..LYRKYIEYPVLQ
Glttk ....MGKSLLRRYLDYPVLW
TM1b TM2a TM2b
50 60 70 80 90 100
EAAT2 LTVFGVILGAVCGGLLRLASP...IHPDVVMLIAFPGDILMRMLKMLILPLIISSLITGL
EAAT1 LTVTAVIVGTILGFTLRPYR....MSYREVKYFSFPGELLMRMLQMLVLPLIISSLVTGM
EAAT3 STVAAVVLGITTGVLVREHSN...LSTLEKFYFAFPGEILMRMLKLIILPLIISSMITGV
EAAT4 LTVSAVVIGVSLAFALRPYQ....LTYRQIKYFSFPGELLMRMLQMLVLPLIVSSLVTGM
EAAT5 LSVLSVIVGCLLGFFLRTRR....LSPQEISYFQFPGELLMRMLKMMILPLVVSSLMSGL
ASCT1 LTVSGVLAGAGLGAALRG....LSLSRTQVTYLAFPGEMLLRMLRMIILPLVVCSLVSGA
ASCT2 LTVVAVVAGVALGLGVSGAGGALALGPERLSAFVFPGELLLRLLRMIILPLVVCSLIGGA
Gltph KILIGLILGAIVGLILGHYG....YADAVKTYVKPFGDLFVRLLKMLVMPIVFASLVVGA
Glttk KILWGLVLGAVFGLIAGHFG....YAGAVKTYIKPFGDLFVRLLKMLVMPIVLASLVVGA
TM3
110 120 130 140 150
EAAT2 SGLDAKASGRLGTRAMVYYMSTTIIAAVLGVILVLAIHPGNPKLKKQLG .....PGKKN
EAAT1 AALDSKASGKMGMRAVVYYMTTTIIAVVIGIIIVIIIHPG.KGTKENMH .....REGKI
EAAT3 AALDSNVSGKIGLRAVVYYFCTTLIAVILGIVLVVSIKPGVTQKVGEIA .....RTGST
EAAT4 ASLDNKATGRMGMRAAVYYMVTTIIAVFIGILMVTIIHPG.KGSKEGLH .....REGRI
EAAT5 ASLDAKTSSRLGVLTVAYYLWTTFMAVIVGIFMVSIIHPG.SAAQKETT .....EQSGK
ASCT1 ASLDASCLGRLGGIAVAYFGLTTLSASALAVALAFIIKPGSGAQTLQSSDLGLEDSGPPP
ASCT2 ASLDPGALGRLGAWALLFFLVTTLLASALGVGLALALQPGAASAAINAS.VGAAGSAENA
Gltph ASISPARLGRVGVKIVVYYLLTSAFAVTLGIIMARLFNPGAGIHLAVGG ....QQ
Glttk ASISPARLGRVGVKIVVYYLATSAMAVFFGLIVGRLFNVGANVNLGSGT.........GK
TM4b
TM4a
160 170 180 190 200
EAAT2 DEVSSLDAFLDLIRNLFPENLVQACFQQIQTVTKKVLVAPPPDEEANATS.........
EAAT1 VRVTAADAFLDLIRNMFPPNLVEACFKQFKTNYEKRSFKVPIQANETLVG.........
EAAT3 PEVSTVDAMLDLIRNMFPENLVQACFQQYK..TKREEVKPPSDPEMNMTE.........
EAAT4 ETIPTADAFMDLIRNMFPPNLVEACFKQFKTQYSTRVVTRTMVRTENGSEPGASMPPPFS
EAAT5 PIMSSADALLDLIRNMFPANLVEATFKQYRTKTTP.VVKSPKVAPEEAPPRRILIYG...
ASCT1 VPKETVDSFLDLARNLFPSNLVVAAFRTYATDYKVVTQNSSSGNV.............
ASCT2 PSKEVLDSFLDLARNIFPSNLVSAAFRSYSTTYEERNITG..................
Gltph FQPKQAPPLVKILLDIVPTNPFGALANGQVLPTIFFAIILG.................
Glttk AIEAQPPSLVQTLLNIVPTNPFASLAKGEVLPVIFFAIILG.................
TM4c
210 220 230 240 250 260
EAAT2 ........AVVSLLNETVTEVPEETKMVIKKGLEFKDGMNVLGLIGFFIAFGIAMGKMGD
EAAT1 .....AVINNVSEAMETLTRIT..EELVPV..PGSVNGVNALGLVVFSMCFGFVIGNMKE
EAAT3 .........ESFTAVMTTAISKNKTKEYKIVG..MYSDGINVLGLIVFCLVFGLVIGKMGE
EAAT4 VENGTSFLENVTRALGTLQEMLSFEETVPV..PGSANGINALGLVVFSVAFGLVIGGMKH
EAAT5 .....VQEENGSHVQNFALDLTPPPEVVYKSEPGTSDGMNVLGIVFFSATMGIMLGRMGD
ASCT1 ....THEKIPIG..TEIEGMNILGLVLFALVLGVALKKLGS
ASCT2 ....TRVKVPVG..QEVEGMNILGLVVFAIVFGVALRKLGP
Gltph ....IAITYLMNSENEKVRK
Glttk ....IAITYLMNRNEERVRK
TM5a TM5b
270 280 290 300 310 320
EAAT2 QAKLMVDFFNILNEIVMKLVIMIMWYSPLGIACLICGKIIAIKDLEVVARQLGMYMVTVI
EAAT1 QGQALREFFDSLNEAIMRLVAVIMWYAPVGILFLIAGKIVEMEDMGVIGGQLAMYTVTVI
EAAT3 KGQILVDFFNALSDATMKIVQIIMCYMPLGILFLIAGKIIEVEDWEIFR.KLGLYMATVL
EAAT4 KGRVLRDFFDSLNEAIMRLVGIIIWYAPVGILFLIAGKILEMEDMAVLGGQLGMYTLTVI
EAAT5 SGAPLVSFCQCLNESVMKIVAVAVWYFPFGIVFLIAGKILEMDDPRAVGKKLGFYSVTVV
ASCT1 EGEDLIRFFNSLNEATMVLVSWIMWYVPVGIMFLVGSKIVEMKDIIVLVTSLGKYIFASI
ASCT2 EGELLIRFFNSFNEATMVLVSWIMWYAPVGIMFLVAGKIVEMEDVGLLFARLGKYILCCL
Gltph SAETLLDAINGLAEAMYKIVNGVMQYAPIGVFALIAYVMAEQG..VKVVGELAKVTAAVY
Glttk SAETLLRVFDGLAEAMYLIVGGVMQYAPIGVFALIAYVMAEQG..VRVVGPLAKVVGAVY

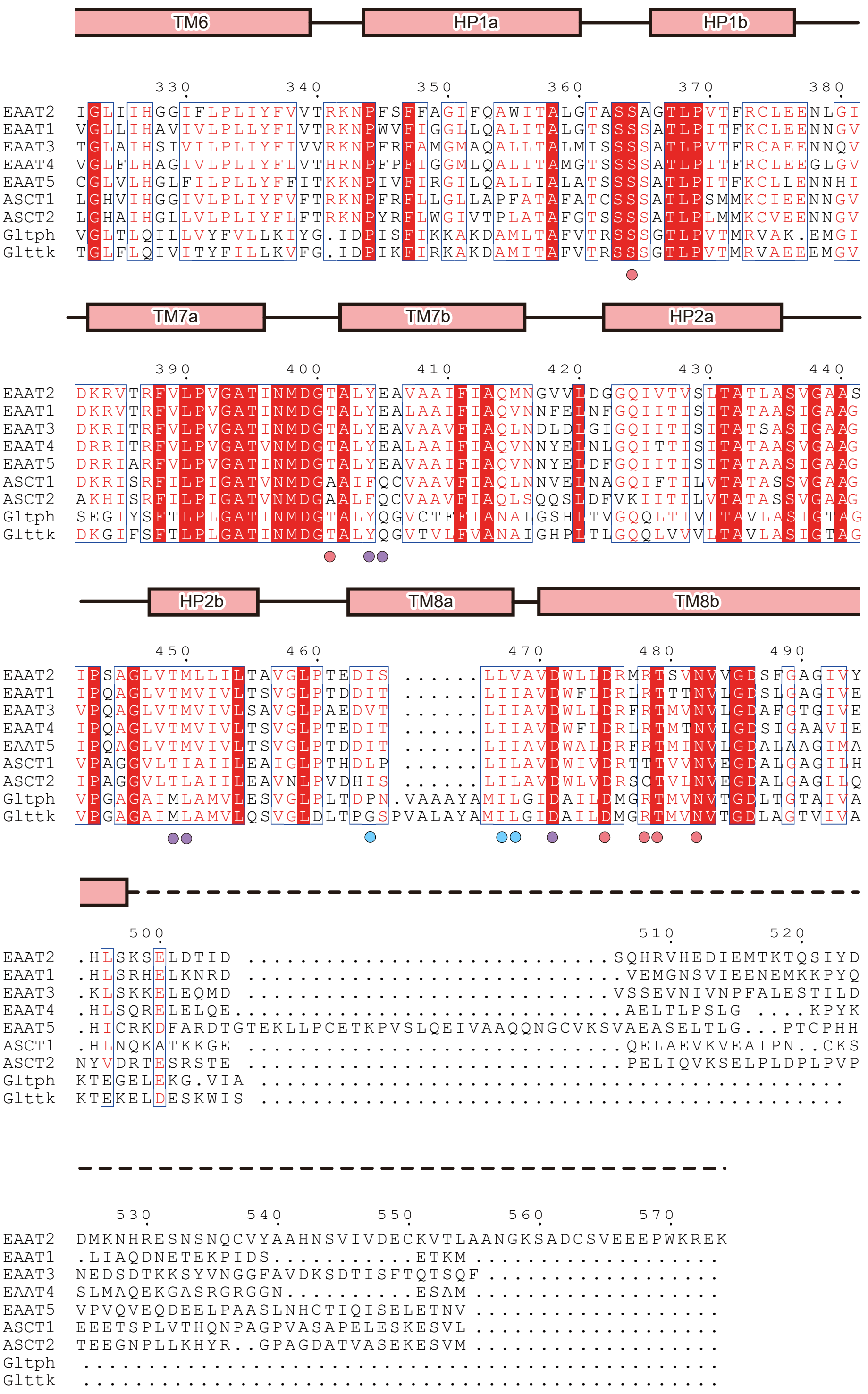

Supplementary Figure 2 | Multiple amino acid sequence alignment of EAAT2

Sequence alignment of *Homo sapiens* EAAT2 (HsEAAT2: UniProt ID P43004), *Homo sapiens* EAAT1 (P43003), *Homo sapiens* EAAT3 (P43005), *Homo sapiens* EAAT4 (P48664), *Homo sapiens* EAAT5 (O00341), *Homo sapiens* ASCT1 (P43007), *Homo sapiens* ASCT2 (Q15758), *Pyrococcus horikoshii* Glt$_{ph}$ (O59090) and *Thermococcus kodakarensis* Glt$_{tk}$ (Q5JID0). The secondary structure of HsEAAT2 is indicated above the sequence. The α-helices, β-strands and disorded regions are indicated by cylinders, arrows and dashed lines, and the scaffold domain and the transport domain are colored light blue and light red, respectively. At the scaffold domain, blue and green triangles indicate kink-inducing residues and Trp286, respectively. At the transport domain, red, purple and blue circles indicate residues of the glutamate-binding site, the cavity the tip around WAY213613, respectively.

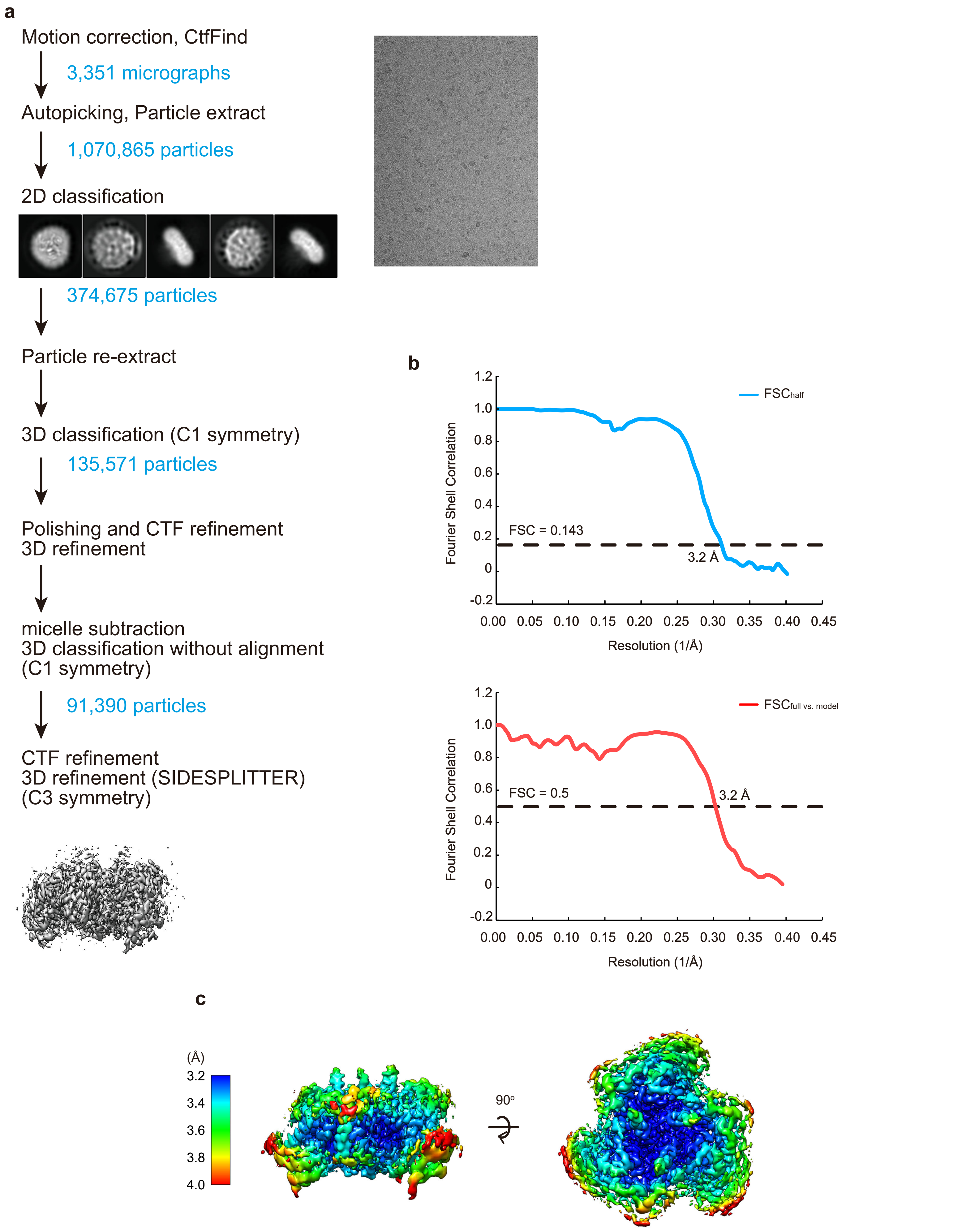

Supplementary Figure 3│cryo-EM analysis of substrate-free HsEAAT2

a, Flow chart of cryo-EM data processing of HsEAAT2 substrate-free structure. **b**, Fourier Shell Correlation (FSC) curve of the final 3D reconstruction model calculated using “relion_postprocess” with masked marked 3.2 Å resolution, corresponding to the FSC = 0.143 gold standard cut-off criterion (blue). FSC curve of map vs. model (red). **c**, Local resolution of the HsEAAT2 substrate-free structure, estimated by RELION.

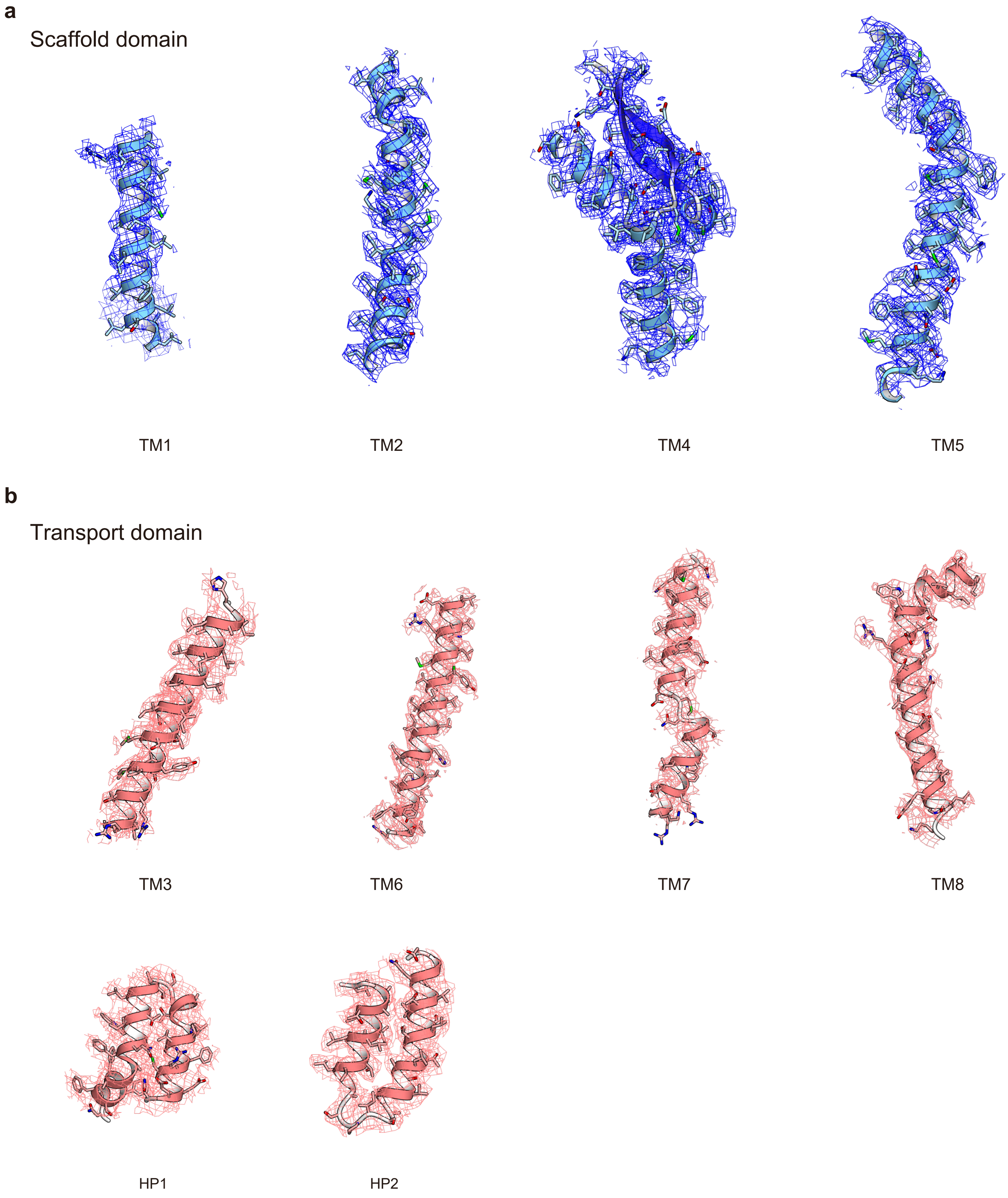

Supplementary Figure 4 │ Atomic model of HsEAAT2 in the cryo-EM map

(**a**, **b**) Densities of all transmembrane helices and helical hairpins (countoured at 1.0σ). **a**, The densities of the scaffold domain, and **b**, the densities of the transport domain and helical hairpins (HP1 and HP2). TM2, TM5, TM7 and TM8 are each diveded into two segments.

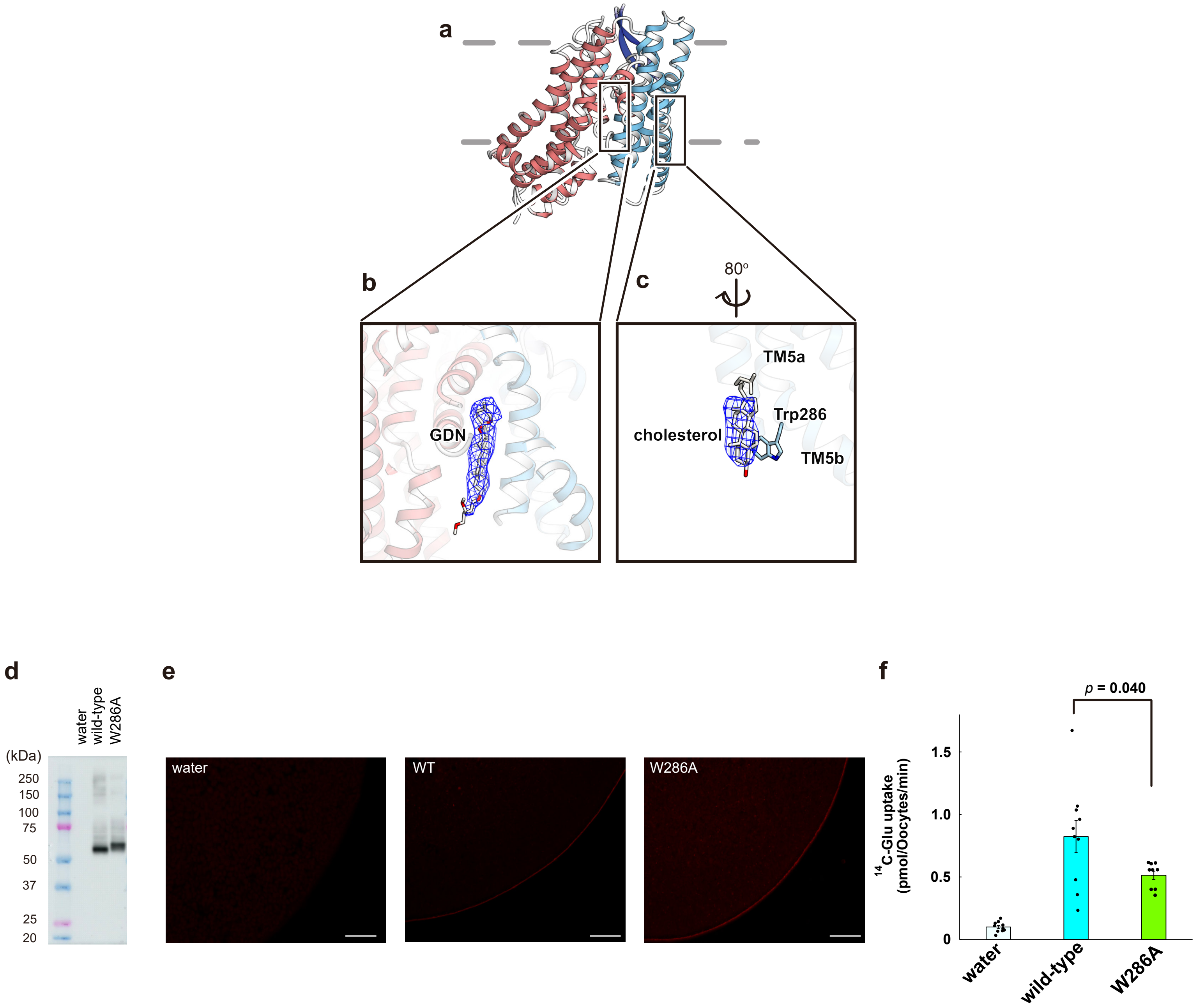

Supplementary Figure 5│Lipid densities of the HsEAAT2 protomer

a, Overall structure of the HsEAAT2 protomer, viewed from the same orientation as in Fig. 2**a**. **b**, **c**, Close-up views of lipid densities. **b**,Density located between the transport domain and the scaffold domain. **c**, Density located at the scaffold domain. $F_o - F_c$ omit maps of both GDN and cholesterol are contoured at 2.5 σ (normalized within mask), and shown as a blue mesh. **d, e** Western blotting and localization (scale bars = 50 μm) of wild type and W286A. **f**, Glutamate uptake of control (*n* = 10), wild type (*n* = 10) and W286A (*n* = 9). Values are mean ± s.e.m. (*P* value is from two-side *t* test. **p* = 0.040. 95% confidence interval).

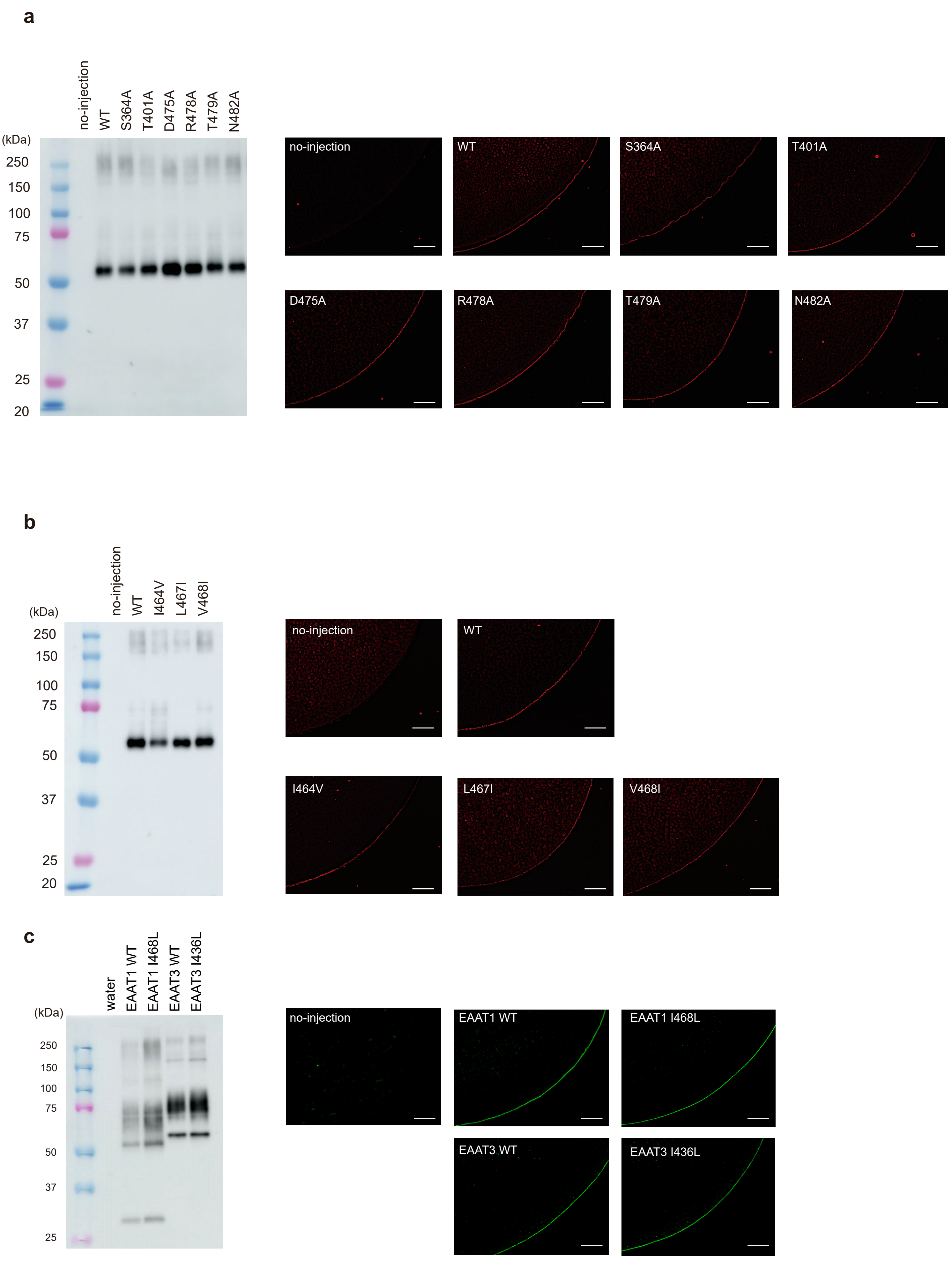

Supplementary Figure 6 | Expression level and localization of each mutant

Expression level and localization for **a**) each mutant of the substrate-binding site, **b**) each mutant of the cavity and **c**) mutants of EAAT1 and EAAT3. The western blotting analysis and the fluorescence detection (scale bars = 50 μm) at the plasma membrane are shown on the left and right sides, respectively. All measurements were repeated at least three times, and one representative result for each mutant is shown.

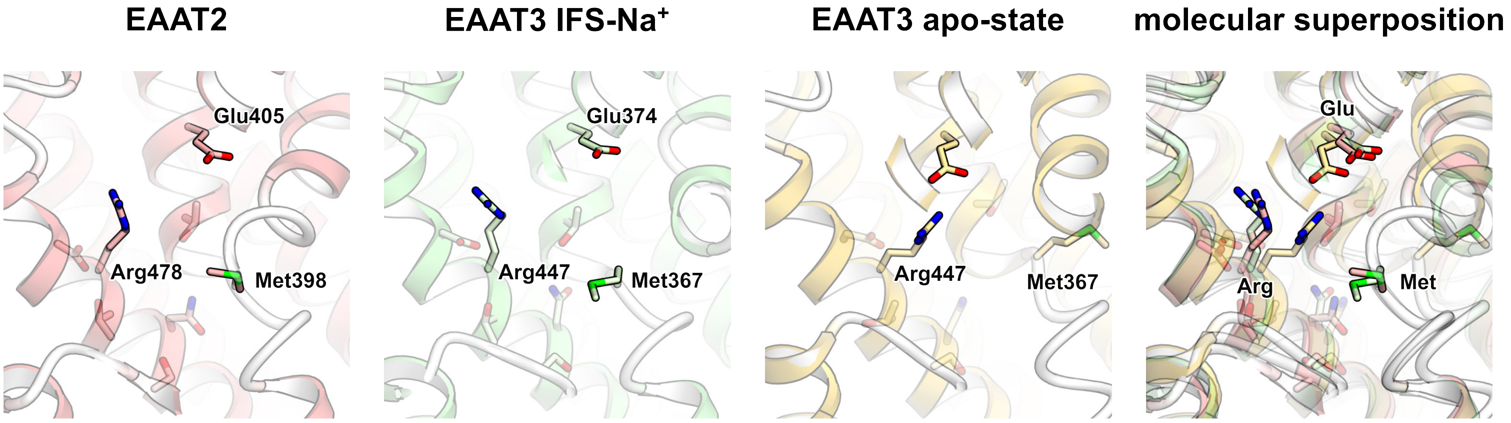

Supplementary Figure 7 | Comparison between EAAT2 and EAAT3

Close-up views of the pockets of EAAT2 substrate-free state, EAAT3 apo-state (PDB ID 6X3F) and EAAT3 IFS-Na^+ (6X2Z), and their molecular superposition. Opaque sidechains indicate residues recognizing aspartate and glutamate at the binding site.

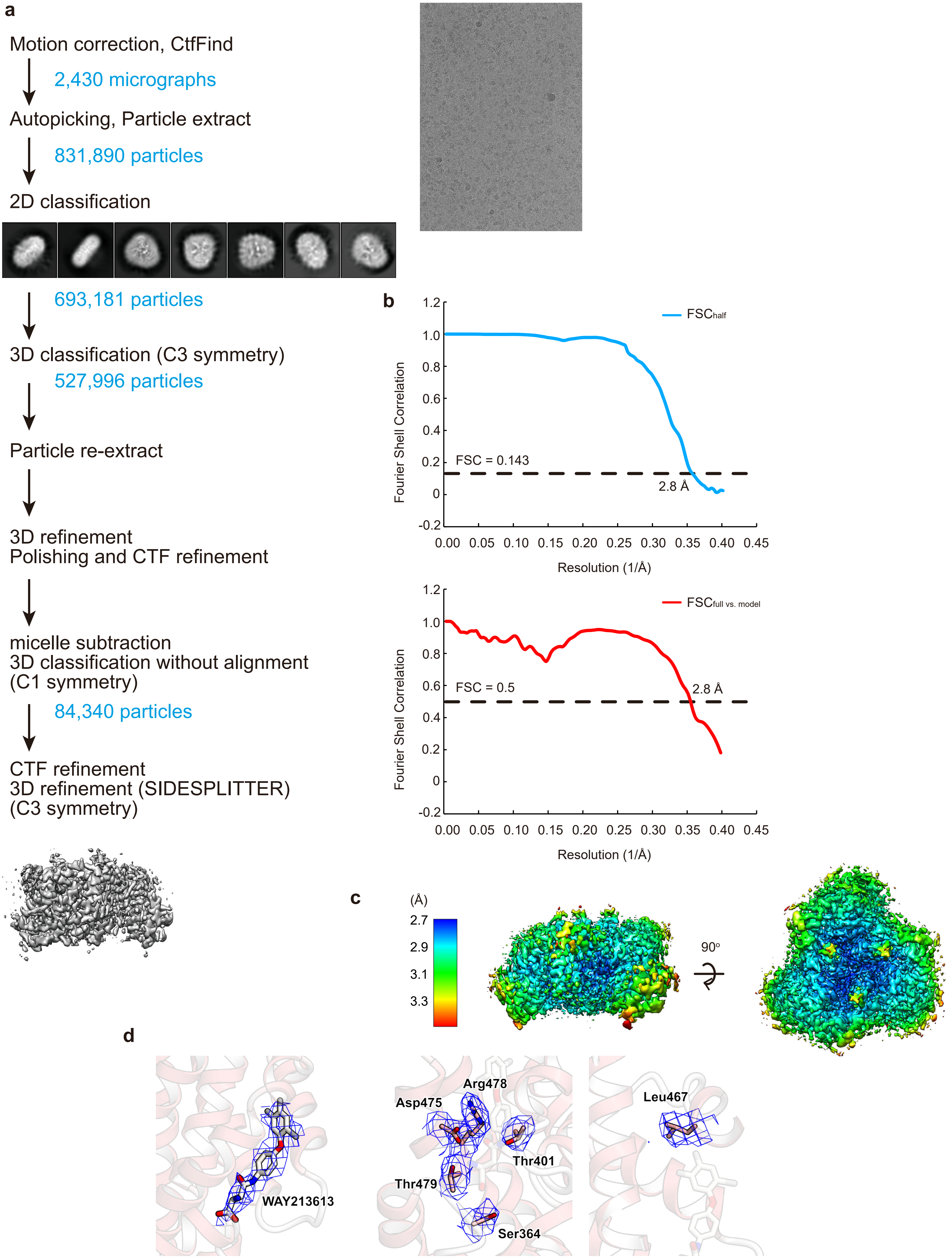

Supplementary Figure 8│cryo-EM analysis of the HsEAAT2 IFS-WAY213613 state

a, Flow chart of cryo-EM data processing of the HsEAAT2 IFS-WAY213613 state. **b**, Fourier Shell Correlation (FSC) curve of the final 3D reconstruction model calculated using “relion_postprocess” with masked marked 2.8 Å resolution, corresponding to the FSC = 0.143 gold standard cut-off criterion (blue). FSC curve of map vs. model (red). **c**, Local resolution of the HsEAAT2 IFS-WAY213613 state. **d**, Densities of WAY213613 (left pannel), residues recognizing the LA moiety (centre pannel) and Leu467 (right pannel).

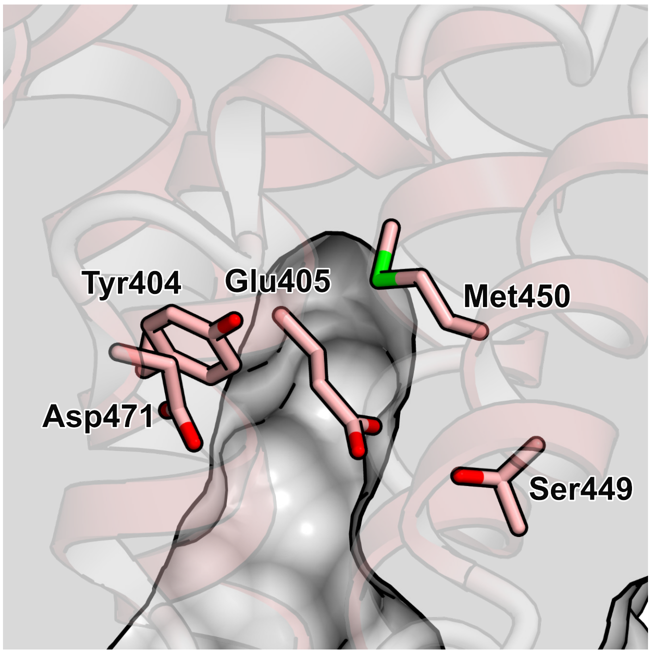

Supplementary Figure 9 │ Conserved residues at the cavity

Close-up view of the cavity formed near the substrate-binding site. Five residues (Tyr404, Glu405, Ser449, Met450 and Asp471) are completely conserved among EAATs.

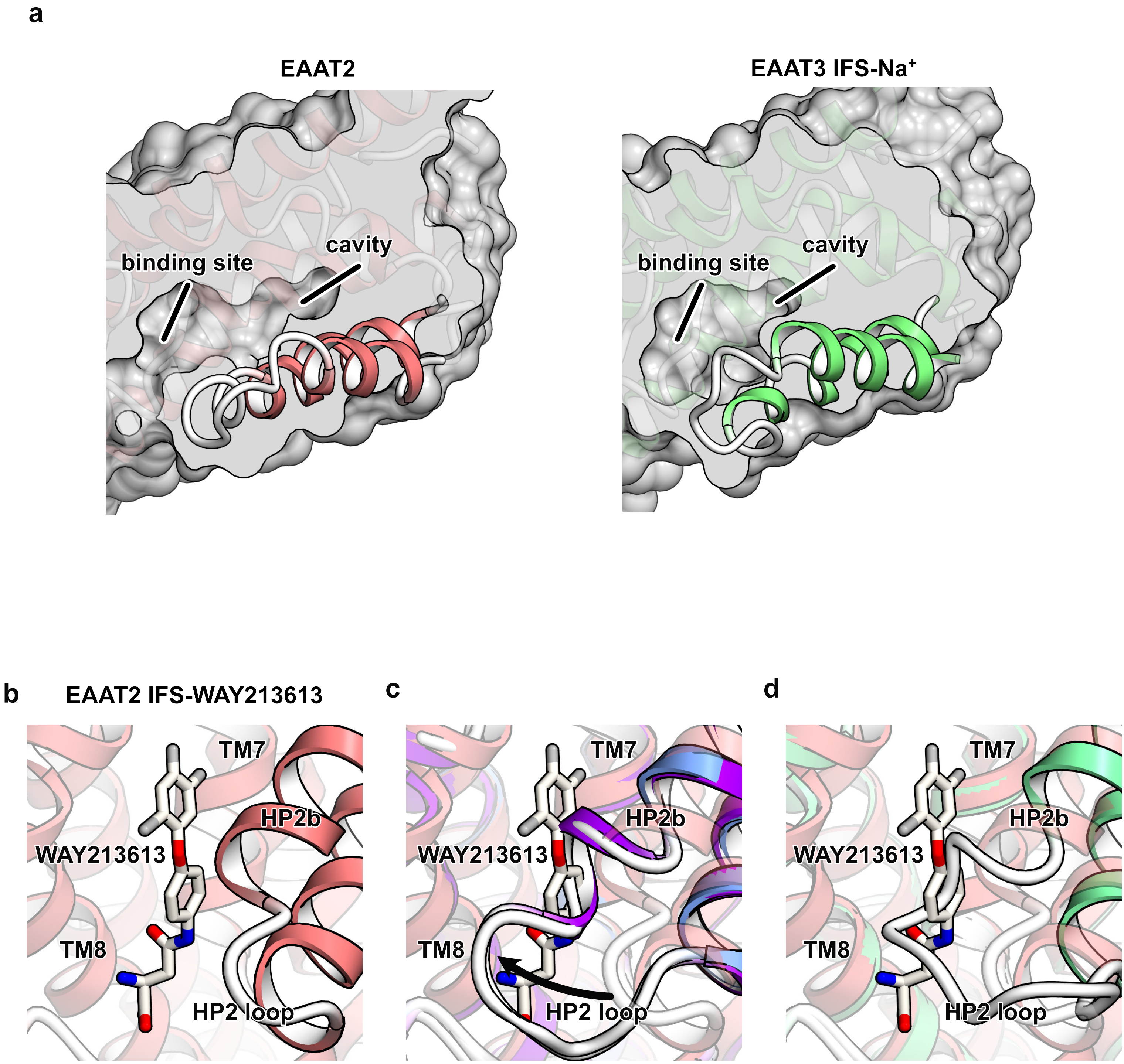

Supplementary Figure 10│Structural comparison of transport domains between HsEAAT2 and other SLC1A transporters

a, Cut-away representations of transport domains. The slab thicknesses are the same. **b,** Close-up view of the WAY-213613 binding site. **c**, Molecular superpositions among the IFS-WAY213613 state and Asp-bound states of EAAT1 (purple and PDB ID 5LLU) and EAAT3 (light blue and 6X2Z). The arrow indicates the movement of the HP2 loop. **d**, Molecular superpositions between IFS-WAY213613 and TFB-TBOA bound state of EAAT1 (green and PDB ID 5MJU).

Supplementary Table 1 │ cryo-EM data collection, model refinement and validation statistics

Cryo-EM data collection, refinement and validation statistics

	HsEAAT2 substrate-free state (EMDB-32098) (PDB 7VR8)	HsEAAT2 IFS-WAY213613 (EMDB-32097) (PDB 7VR7)
**Data collection and processing**		
Magnification	×105,000	×105,000
Voltage (kV)	300	300
Electron exposure (e–/Å^2)	50	50
Defocus range (μm)	-0.8 to -1.6	-0.8 to -1.6
Pixel size (Å)	0.83	0.83
Symmetry imposed	*C*3	*C*3
Initial particle images (no.)	1,090,865	831,890
Final particle images (no.)	91,390	84,340
Map resolution (Å)	3.2	2.8
FSC threshold	0.143	0.143
**Refinement**		
Initial model used (PDB code)	6GCT	
Model composition in the asymmetric unit		
Non-hydrogen atoms	3,012	3,012
Protein residues	402	402
Ligands[a]	4	6
Average *B* factors (Å^2)		
Protein	173.8	88.9
Ligand	221.4	143.0
R.m.s. deviations		
Bond lengths (Å)	0.017	0.015
Bond angles (°)	2.05	2.02
Validation		
MolProbity score	2.45	2.52
Clashscore	9.95	9.62
Poor rotamers (%)	5.79	7.01
Ramachandran plot		
Favored (%)	94.92	94.67
Allowed (%)	5.08	7.5.33
Outlier (%)	0	0

[a] Ligands include cholesterol, head moiety of GDN, phospholipid and WAY213613

Uncropped scans of blots in Supplementary Figures

Supplementary Figures 5d

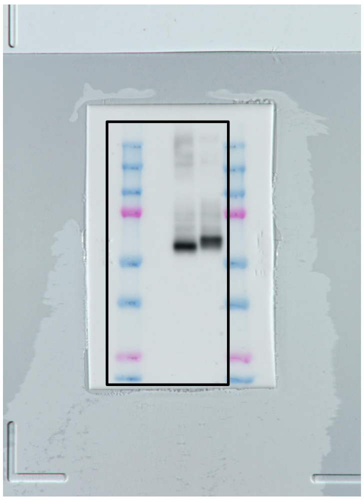

Supplementary Figures 6

a

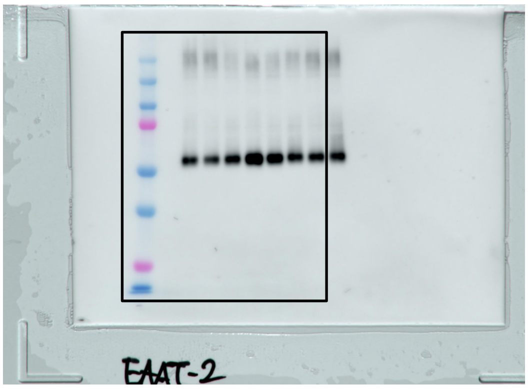

b

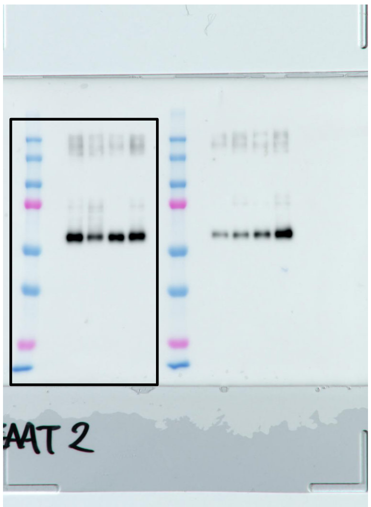

c

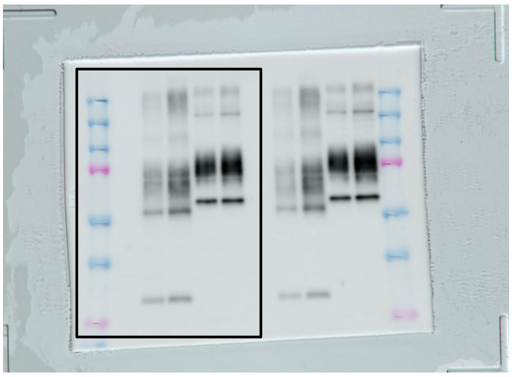

Supplement: Supplementary file 1 — Supplementary Information [file 41467_2022_32442_MOESM1_ESM.pdf]
