# Supplementary material for: Structural insights into inhibitory mechanism of human excitatory amino acid transporter EAAT2
Source: Nat Commun. 2022 Aug 11;13:4714. doi: 10.1038/s41467-022-32442-6 (PMC9372063; doi:10.1038/s41467-022-32442-6)
Supplement: Supplementary file 3 — Reporting Summary [file 41467_2022_32442_MOESM3_ESM.pdf]

nature portfolio

Corresponding author(s): Tsukasa Kusakizako
Yoshikatsu Kanai
Osamu Nureki

Last updated by author(s): Jul 15, 2022

Reporting Summary

Nature Portfolio wishes to improve the reproducibility of the work that we publish. This form provides structure for consistency and transparency in reporting. For further information on Nature Portfolio policies, see our Editorial Policies and the Editorial Policy Checklist.

Statistics

For all statistical analyses, confirm that the following items are present in the figure legend, table legend, main text, or Methods section.

n/a	Confirmed	
☐	☒	The exact sample size (*n*) for each experimental group/condition, given as a discrete number and unit of measurement
☐	☒	A statement on whether measurements were taken from distinct samples or whether the same sample was measured repeatedly
☐	☒	The statistical test(s) used AND whether they are one- or two-sided<br>*Only common tests should be described solely by name; describe more complex techniques in the Methods section.*
☒	☐	A description of all covariates tested
☒	☐	A description of any assumptions or corrections, such as tests of normality and adjustment for multiple comparisons
☐	☒	A full description of the statistical parameters including central tendency (e.g. means) or other basic estimates (e.g. regression coefficient) AND variation (e.g. standard deviation) or associated estimates of uncertainty (e.g. confidence intervals)
☐	☒	For null hypothesis testing, the test statistic (e.g. *F*, *t*, *r*) with confidence intervals, effect sizes, degrees of freedom and *P* value noted<br>*Give P values as exact values whenever suitable.*
☒	☐	For Bayesian analysis, information on the choice of priors and Markov chain Monte Carlo settings
☒	☐	For hierarchical and complex designs, identification of the appropriate level for tests and full reporting of outcomes
☒	☐	Estimates of effect sizes (e.g. Cohen's *d*, Pearson's *r*), indicating how they were calculated

Our web collection on statistics for biologists contains articles on many of the points above.

Software and code

Policy information about availability of computer code

Data collection	SerialEM 3.7.4
Data analysis	MotionCor2, CTFFIND 4.1, RELION3.1, SIDESPLITTER, PHENIX-1.143260, WinCoot 0.8.9.2, CueMol2.2.3.443, Chimera 1.13.1, Phyre2, Refmac5, Servalcat, PROPKA, Microsoft Excel and BOILED-Egg

For manuscripts utilizing custom algorithms or software that are central to the research but not yet described in published literature, software must be made available to editors and reviewers. We strongly encourage code deposition in a community repository (e.g. GitHub). See the Nature Portfolio guidelines for submitting code & software for further information.

Data

Policy information about availability of data

All manuscripts must include a data availability statement. This statement should provide the following information, where applicable:

- Accession codes, unique identifiers, or web links for publicly available datasets
- A description of any restrictions on data availability
- For clinical datasets or third party data, please ensure that the statement adheres to our policy

We included accession codes of cryo-EM maps and structures of human EAAT2 (EMD-32098 (the substrate-free state) and EMD-32097 (the IFS-WAY213613 state). The PDB accession codes for the coordinates are 7VR8 (the substrate-free state) and 7VR7 (the IFS-WAY213613 state)). The raw images have been deposited in the Electron Microscopy Public Image Archive, under accession code EMPIAR-11084. These information are in the section "Data availability".

Field-specific reporting

Please select the one below that is the best fit for your research. If you are not sure, read the appropriate sections before making your selection.

☒ Life sciences ☐ Behavioural & social sciences ☐ Ecological, evolutionary & environmental sciences

For a reference copy of the document with all sections, see nature.com/documents/nr-reporting-summary-flat.pdf

Life sciences study design

All studies must disclose on these points even when the disclosure is negative.

Sample size	No statistical methods were used to predetermine the sample size. For cryo-EM analyses, sample sizes were determined by the availability of microscope sessions, and the number of particles on electron microscopy grids were enough to obtain a structure at the reported resolution. In oocytes assays, we used 4-10 oocytes expressing each construct and performed transport assays. Each result are monitored by 3 trials (multiple Xenopus batches and days) at least.
Data exclusions	For cryo-EM processing, particles which did not contribute to map quality were excluded following the standard classification procedures. This is standard practice for structure determination by cryo-EM. For oocyte assays, all measurements were repeated at three times (different batches), and one representative trace for each mutant is shown.
Replication	For cryo-EM analyses, related experiments including FSEC, purification, and SDS-PAGE were reproduced at three times at least and structure determination was completed once. For oocyte assays, all data sets were pooled from at least three independent Xenopus batches.
Randomization	For cryo-EM analyses, particles were randomly assigned to half-maps for resolution determination following the standard procedures in RELION. For oocyte assays, randomization was not performed since samples were not divided into two or more groups.
Blinding	For cryo-EM analyses, blinding was not applicable since this type of studies does not use group allocation. For oocyte assays, blinding was not applied since it was not technically or practically feasible to do so.

Reporting for specific materials, systems and methods

We require information from authors about some types of materials, experimental systems and methods used in many studies. Here, indicate whether each material, system or method listed is relevant to your study. If you are not sure if a list item applies to your research, read the appropriate section before selecting a response.

Materials & experimental systems

n/a	Involved in the study	
☐	☒	Antibodies
☐	☒	Eukaryotic cell lines
☒	☐	Palaeontology and archaeology
☐	☒	Animals and other organisms
☒	☐	Human research participants
☒	☐	Clinical data
☒	☐	Dual use research of concern

Methods

n/a	Involved in the study	
☒	☐	ChIP-seq
☒	☐	Flow cytometry
☒	☐	MRI-based neuroimaging

Antibodies

Antibodies used	rabbit anti-human EAAT2 antibody (cat no sc-365634, Santa Cruz Biotechnology), rabbit anti-HA antibody (cat no 561, MBL Life Science), peroxidase goat anti-rabbit IgG (AB_2307391, Jackson ImmunoResearch) and Alexa Fluor 488-conjugated anti-rabbit IgG (A21206, Invitrogen)
Validation	rabbit anti-human EAAT2 antibody (cat no sc-365634, Santa Cruz Biotechnology): https://www.sigmaaldrich.com/GB/en/product/sigma/hpa009172?gclid=CjwKCAjwh-CVBhB8EiwAjFEPGblQG6jezTdX_bRWdHvHhg5PJusDO2KRTaxXEHXVLGwz0tSyiA-sRRoC0KIQAvD_BwE<br>rabbit anti-HA antibody (cat no 561, MBL Life Science): https://www.mblbio.com/bio/g/dtl/A/?pcd=561<br>peroxidase goat anti-rabbit IgG (AB_2307391, Jackson ImmunoResearch): https://www.jacksonimmuno.com/catalog/products/111-035-144<br>Alexa Fluor 488-conjugated anti-rabbit IgG (A21206, Invitrogen): https://www.thermofisher.com/antibody/product/Donkey-anti-Rabbit-IgG-H-L-Highly-Cross-Adsorbed-Secondary-Antibody-Polyclonal/A-21206

Eukaryotic cell lines

Policy information about cell lines

Cell line source(s)	HEK293S GnTI- (ATCC, Cat.#CRL-3022), Sf9 (ATCC, Cat.#CRL-1711)
Authentication	The cell lines listed above were purchased from ATCC Cell lines and no further authentication was performed.
Mycoplasma contamination	Not tested (Sf9 and HEK293 cell lines); tested for negative (HEK293) using MycoAlert Mycoplasma detection kit (Lonza)
Commonly misidentified lines (See ICLAC register)	HEK cells are listed in the register but it does not specify which type of HEK strains. Our secondary HEL293S GnTI- cell lines was purchased by from ATCC, where they validated.

Animals and other organisms

Policy information about studies involving animals; ARRIVE guidelines recommended for reporting animal research

Laboratory animals	Xenopus laevis
Wild animals	This study did not use wild animals.
Field-collected samples	This study did not use field-collected samples.
Ethics oversight	This study did not use materials that require ethical approval.

Note that full information on the approval of the study protocol must also be provided in the manuscript.
