# Supplementary material for: Structural insights into inhibitory mechanism of human excitatory amino acid transporter EAAT2
Source: Nat Commun. 2022 Aug 11;13:4714. doi: 10.1038/s41467-022-32442-6 (PMC9372063; doi:10.1038/s41467-022-32442-6)
Supplement: Supplementary file 2 — Peer Review File [file 41467_2022_32442_MOESM2_ESM.pdf]

nature portfolio

Peer Review File

Structural insights into inhibitory mechanism of human excitatory amino acid transporter EAAT2

Open Access This file is licensed under a Creative Commons Attribution 4.0 International License, which permits use, sharing, adaptation, distribution and reproduction in any medium or format, as long as you give appropriate credit to the original author(s) and the source, provide a link to the Creative Commons license, and indicate if changes were made. In the cases where the authors are anonymous, such as is the case for the reports of anonymous peer reviewers, author attribution should be to 'Anonymous Referee' followed by a clear attribution to the source work. The images or other third party material in this file are included in the article's Creative Commons license, unless indicated otherwise in a credit line to the material. If material is not included in the article's Creative Commons license and your intended use is not permitted by statutory regulation or exceeds the permitted use, you will need to obtain permission directly from the copyright holder. To view a copy of this license, visit http://creativecommons.org/licenses/by/4.0/.

REVIEWER COMMENTS</B>

Reviewer #1 (Remarks to the Author):

Kato et. al. report human EAAT2 structures determined in Apo and inhibitor-bound states. Despites that many structures of EAATs were reported recently, these two structures could still enhance our knowledge about these therapeutic drug targets.

My major concern is that the cryo-EM reconstruction appears to have room for improvement.

First, there is a white line in both micrographs (Supplementary Figure 3 and 8), suggesting that the gain reference may need be flipped horizontally (relion_image_handler --i gain.mrc --o gain_flipY.mrc --flipY).

Second, the micrographs look excellent and > 200 K particles were used for final reconstructions. A further 3D classification without 3-fold symmetry may improve resolution.

Minor comments:

1. Could the authors also show the cryo-EM density for the inhibitor?

2. In Figure 2 legend, it should read “kink-inducing?”

3. In Figure 3, could the authors add statistic?

4. In Figure 2d, it is confusing that TM2 is not shown, but it contributes to trimerization based on the text.

5. In Figure 3, could the authors highlight polar interactions?

6. Please also provide FSC for map versus model.

7. In Figure 5, it may not be possible to distinguish GDN and cholesterol based on the maps of 3.4 angstrom resolution.

Reviewer #2 (Remarks to the Author):

SUMMARY

In this manuscript, Kato et al. solve the structures of human EAAT2 in an apo and inhibitor-bound structure using cryo-EM. The inhibitor is selective for EAAT2 over other EAATs and is called WAY-213613. Additionally, residues that coordinate WAY-213613 in EAAT2 to the equivalent residue in other EAATs that are less sensitive to the inhibitor to delineate a structure function activity relationship. The manuscript is generally well written, and the figures clearly represent the data.

MAJOR COMMENTS

1. Lines 74-75: If deficiencies in EAAT2 cause disease, then how will inhibitors of EAAT2 be clinically useful? If targeting to cancer cells is required, this reviewer thinks this should be explicitly started as inhibition of EAAT2 for clinical benefit comes with high risk of excitotoxicity.

2. Cholesterol modulation of EAT2 function is a very interesting concept. I would be nice to see the effect of mutating residues that coordinate cholesterol (Trp286 and others) on transporter function.

3. While the functional data for mutations in the inhibitor binding site is appreciated, Full dose response curves with a minimum of 7 concentrations of inhibitor should be used to properly delineate the effect of the mutagenesis on inhibition by WAY-213613. In line with this, saying that “inhibition by WAY-213613 was more or less affected by all three mutants” is too vague. Further data and interpretation are required.

MINOR COMMENTS

• Lines 52-53: “although excessive glutamate at synaptic clefts is associated with greater calcium influx.” Is a confusing end to this sentence – please edit for clarity.

• Lines 56-57: “Especially, EAAT2 …. is highly expressed at the plasma membrane of glial cells” should be replace with “EAAT2 …. is particularly highly expressed at the plasma membrane of glial cells”

• Lines 182-183: “a similar arrangement of the substrate-binding site could be observed in the outward-facing state of HsEAAT2.” Saying “could be” implies there is data for this which there is not. Please replace with “may be” or “is likely to be”.

Reviewer #3 (Remarks to the Author):

The manuscript by Kato et al. describes cryo-EM atomic models of human excitatory neurotransmitter transporter EAAT2 (SLC1A2) in the absence of substrate, as well as in the presence of a -commercially available- competitive inhibitor, WAY213613. A few functional experiments, with transporters heterologously expressed in oocytes, accompany the structural results.

Overall, the manuscript suffers from lack of novelty and insights on transport and/or pharmacological mechanisms of excitatory amino acid transporters. The work appears technically correct, but neither structural nor functional experiments are particularly challenging.

Major concerns:

1-There have been already several reports of experimentally-determined structures of human members of the SLC1A family (EAAT1, EAAT3, and ASCT2), and although the manuscript by Kato et al. reports the first structures of EAAT2, they lack new insights on the transport mechanism of the family. Inward-facing EAAT2 structure in the absence substrate (Fig. 1 and 2) nearly resembles the recently reported structure of EAAT3 (Qui et al., Sci Adv), but it is of lower resolution (~3.5 compared to 2.9 to angstroms). As the authors show in the manuscript, there are no "surprises" in the EAAT2 structure compared to published structural work on SLC1A proteins.

2-The authors assign extra cryo-EM density to GDN and cholesterol molecules (Supp. Fig. 5). However, the density blobs shown in that fig. do not appear to have molecular features to unambiguously assign those molecules. Furthermore, there is no functional-experimental effort whatsoever to test effects of lipids, in particular cholesterol, and the authors rely on published work by others to proposed vague modulatory roles of lipids on transport function.

3-The molecular mechanism underlying competitive inhibition in this family of transporters, whereby the inhibitor precludes movements of HP2, was nicely demonstrated by the Gouaux laboratory back in 2007 (Boudker et al., Nature 2007), and it has been later confirmed in several reports. WAY213613 was developed more >15 years ago, and was then shown to be a competitive glutamate-transport inhibitor using functional assays (Dunlop et al., Mol Pharm, 2005). Hence, the proposed WAY213613 inhibitory mechanism in the manuscript (Fig. 4 and Fig. 6) is just confirmatory.

4-The blob of density corresponding to bound WAY213613 (Fig. 4) seems to lack molecular features to reveal the exact atomic coordination of this molecule, and a more thorough mutagenesis study is necessary to uncover the molecular determinants underlying the ~ 50-fold selectivity of this compound for EAAT2 over EAAT1 and 3 (Dunlop et al., Mol Pharm, 2005). Importantly, in my view, the potential therapeutic use of WAY213613 for cancer therapy in peripheral organs, as suggested in the manuscript, is overshadowed by the risk of a central neurotoxic effect on excitatory synapses. The question remains, if it is really relevant to understand the molecular determinants of WAY213613 selectivity for EAAT2.

In conclusion, the manuscript lacks important mechanistic functional or pharmacological insights on EAATs, and I would not recommend publication.

We appreciate the insightful reviews by the referees and believe we have addressed all of their comments. We have three new conclusions. First, we repeated the data processing, based on Reviewer #1's comments, and improved the resolutions of the substrate-free state and the IFS-WAY213613 state to 3.2 and 2.8 angstroms, respectively. Second, we measured glutamate uptake by the mutant of Trp286, which interacts with endogenous cholesterol molecules. This mutation decreased the glutamate uptake activity, as compared to wild type EAAT2. Third, we conducted WAY213613 inhibition assays for EAAT1 and EAAT3, using their mutants. In our previous manuscript, we suggested that the selectivity of WAY213613 is defined by the cavity environments of the transport domain. The new inhibition assay also supports our insight into this inhibitor selectivity. Therefore, we added Fig. 5**c** and Supplementary Figs. 5**d**, **e**, **f**, **g,** 6**c** and 8**d** and slightly modified Supplementary Figs. 3**b**, **c,** 4, 8**b** and **c**. All changes in the revised manuscript are highlighted in yellow.

Responses to Referees

Reviewer #1 (Remarks to the Author):

Kato et. al. report human EAAT2 structures determined in Apo and inhibitor-bound states. Despites that many structures of EAATs were reported recently, these two structures could still enhance our knowledge about these therapeutic drug targets.

Answer:

We appreciate the kind review and comments on our manuscript. Our point-by-point answers to your comments are described below.

My major concern is that the cryo-EM reconstruction appears to have room for improvement.
First, there is a white line in both micrographs (Supplementary Figure 3 and 8), suggesting that the gain reference may need be flipped horizontally (relion_image_handler --i gain.mrc --o gain_flipY.mrc --flipY).

Thank you for the comment. As Reviewer #1 pointed out, the gain reference was inappropriately flipped in the initial manuscript. Thus, we have generated the new gain reference based on each movie dataset using the 'relion_estimate_gain' subprogram in RELION-3.1, and confirmed that the white line on the micrographs disappeared after the gain correction. We updated the representative micrographs, which are now appropriately gain-corrected (Supplemental Figs. 3 and 8). We applied the new gain reference during Bayesian polishing and re-processed the following steps.

Second, the micrographs look excellent and > 200 K particles were used for final reconstructions. A further 3D classification without 3-fold symmetry may improve resolution.

Thank you for the comment. According to the Reviewer's comment, we performed a further 3D classification without 3-fold symmetry. We selected particles (< 100 K) that show the trimer even with C1 symmetry, contributing to the good reconstruction, for each dataset. Combined with the iterative CTF refinement and the micelle subtraction, our re-processing improved the resolution for each dataset: from 3.6 Å to 3.2 Å (substrate-free state) and from 3.5 Å to 2.8 Å (IFS-WAY213613). The quality of the resulting map, particularly the density of WAY213613, was also improved.

Minor comments:

1. Could the authors also show the cryo-EM density for the inhibitor?

We added the density of WAY213613 in Supplemental Fig. 8. This is not the F_o-F_c map, but the raw density.

2. In Figure 2 legend, it should read "kink-inducing?"

We have corrected it.

3. In Figure 3, could the authors add statistic?

We have added statistics in Fig. 3b.

4. In Figure 2d, it is confusing that TM2 is not shown, but it contributes to trimerization based on the text.

We have added TM2 in Fig. 2d.

5. In Figure 3, could the authors highlight polar interactions?

We have highlighted them with dotted lines.

6. Please also provide FSC for map versus model.

According to the Reviewer's comment, we have included the FSC for map versus model in Sup. Figs. 3**b** and 8**b**.

7. In Figure 5, it may not be possible to distinguish GDN and cholesterol based on the maps of 3.4 angstrom resolution.

Thank you for this comment. The hydrophobic portion of GDN is composed of six aromatic rings, while that of cholesterol is composed of four rings. Accordingly, the former density is larger than the latter one, which is consistent with our improved cryo-EM maps. Moreover, we assigned the head moiety of GDN and the cholesterol molecule, based on the shape and size of their densities (Cover Fig. 1). During the review process of our manuscript, the cryo-EM structure of EAAT1 was reported (*Canul-Tec et al., EMBO 2021*). EAAT1 also has a tryptophan residue in the same position of TM5, and the density resembling cholesterol was observed (Cover Fig. 2). This result is similar to our structure and former ASCT2 studies. Moreover, the cysteine residue that corresponds to Trp286 of HsEAAT2 on the sequence alignment (Supplemental Fig. 2) interacts with a phospholipid in another structure of EAAT3 (Cover Fig. 2) (PDB ID 6S3Q). However, the EAAT3 paper has not been published yet. Considering the similarities and the differences among EAAT2, EAAT1 and EAAT3, we think the conserved tryptophan residues are at least related to the cholesterol binding and have added this information from line 177.

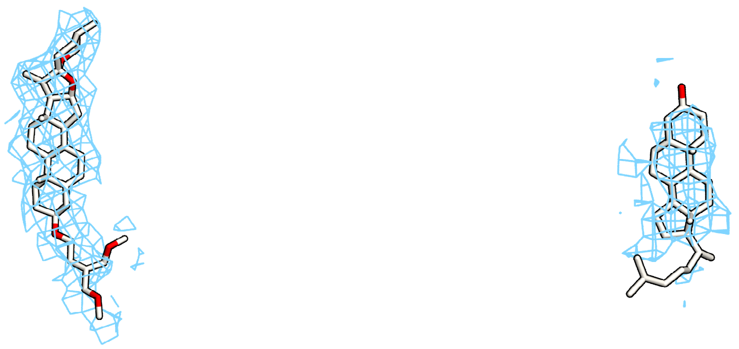

Cover Fig. 1 Density shapes and sizes of GDN and cholesterol

Densities of GDN (left) and cholesterol (right). GDN and cholesterol consist of six and four aromatic rings, respectively, and the shape and size of each density correspond to each molecule.

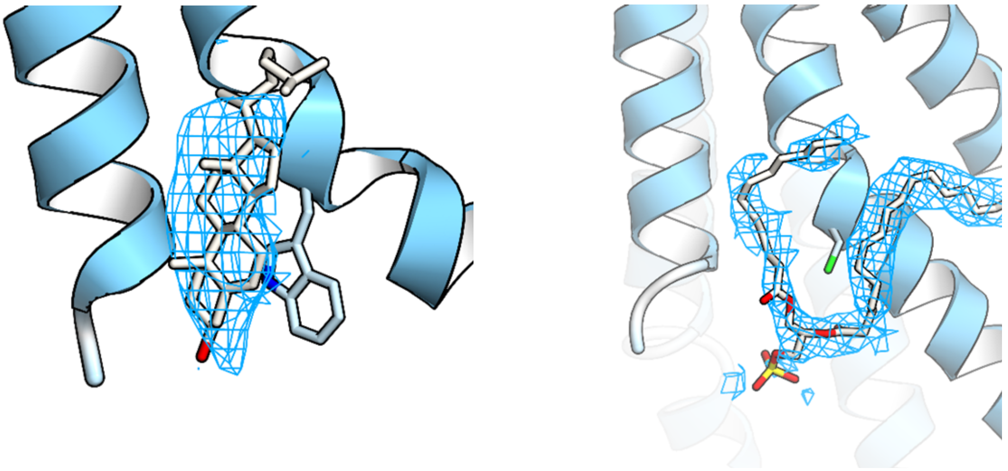

Cover Fig. 2 Cholesterol and lipid interactions with TM5 of EAAT1 and EAAT3

Densities of cholesterol (left) and phospholipid (right) near TM5s of EAAT1 and EAAT3.

Reviewer #2 (Remarks to the Author):

SUMMARY

In this manuscript, Kato et al. solve the structures of human EAAT2 in an apo and inhibitor-bound structure using cryo-EM. The inhibitor is selective for EAAT2 over other EAATs and is called WAY-213613. Additionally, residues that coordinate WAY-213613 in EAAT2 to the equivalent residue in

other EAATs that are less sensitive to the inhibitor to delineate a structure function activity relationship. The manuscript is generally well written, and the figures clearly represent the data.

Answer:

We appreciate the kind review and comments on our manuscript. Our point-by-point responses to your comments are below.

MAJOR COMMENTS

1. Lines 74-75: If deficiencies in EAAT2 cause disease, then how will inhibitors of EAAT2 be clinically useful? If targeting to cancer cells is required, this reviewer thinks this should be explicitly started as inhibition of EAAT2 for clinical benefit comes with high risk of excitotoxicity.

Thank you for this comment. Lower transport activities of EAAT2 are related to excitotoxity, and thus once the inhibitors pass through the blood brain barrier (BBB), there must be some problems. The first research on the relationships between a tumor and EAAT2 was reported 11 years ago, indicating that further investigation regarding the drug efficacy of inhibitors are being required. Although the passage of WAY213613 through the BBB has not been evaluated yet in animal experiments, we assessed its potential by the SwissADME server. This server includes the BOILED-Egg program, which predicts the BBB permeability of compounds based on tPSA (topological polar surface area) versus WLOGP (log P value developed by Wild man and Crippen) plots. The results indicated that WAY213613 could not permeate through the BBB due to its large tPSA (Cover Fig. 3). Whereas this information is a prediction and thus needs to be experimentally validated in future studies to pursue the clinical potential of WAY213613, this is important topics for our present study. Therefore, we have added this information in lines 309.

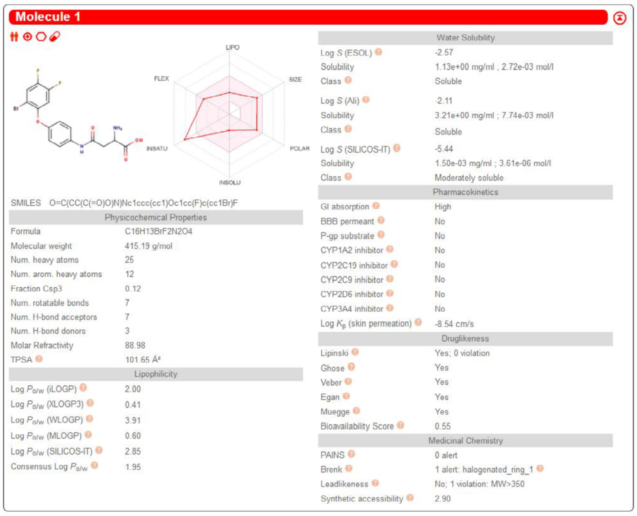

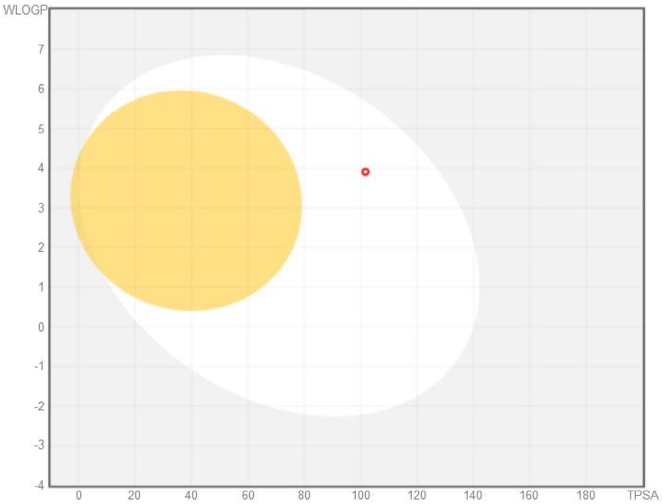

Cover Fig. 3 BBB impermeability of WAY213613

SwissADME results (left). In the Pharmacokinetics session, although "GI (gastrointestinal) absorption" is high, "BBB permeant" shows "No", indicating the BBB impermeability of WAY213613. BOILED-Egg program results (right). This chart demonstrates two values, tPSA (horizontal value: topological polar surface area) and WLOGP (vertical value: log P method developed by Wild- man and Crippen). White and yellow areas correspond to gastrointestinal absorption and BBB permeability, respectively, and the red dot corresponds to WAY213613.

2. Cholesterol modulation of EAT2 function is a very interesting concept. I would be nice to see the effect of mutating residues that coordinate cholesterol (Trp286 and others) on transporter function.

Thank you for this comment. During the review process of our manuscript, the cryo-EM structure of EAAT1 was published. Similar to our results, the tryptophan residue on TM5 also coordinates the cholesterol molecule. Additionally, the unpublished structure of EAAT3 coordinate the phospholipid, while the corresponding residue is cysteine. We have described the former similarity between EAAT2 and EAAT1, starting on line177.

The endogenous cholesterol is captured by only one residue (Trp286). To investigate the role of Trp286, we made a point mutant (W286A) and measured its expression level, localization and transport activity, using *Xenopus* oocytes. The expression level and the plasma membrane localization of the mutant were comparable with those of the wild type (Cover Fig. 4), but the glutamate uptake by W286A was markedly reduced (Cover Fig. 5). This result demonstrates the importance of Trp286 for the transport function of EAAT2, implying the possible contribution of the cholesterol binding to this residue. We have added these results in line 183 and Supplemental Fig. 5**e-g**.

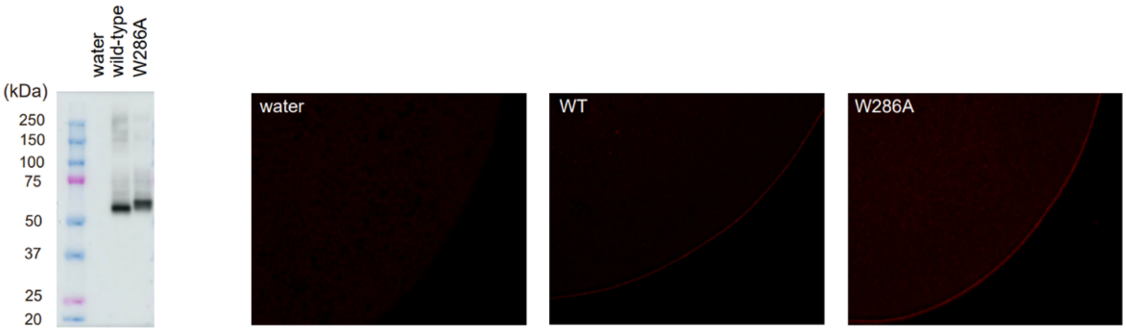

Cover Fig. 4 Expression levels and localizations of the wild type and the W286A mutant of EAAT2

Western blotting (left) and immunofluorescence analyses (right) of wild type EAAT2 and the W286A mutant. Both the expression level and localization of the wild type are similar to those of W286A.

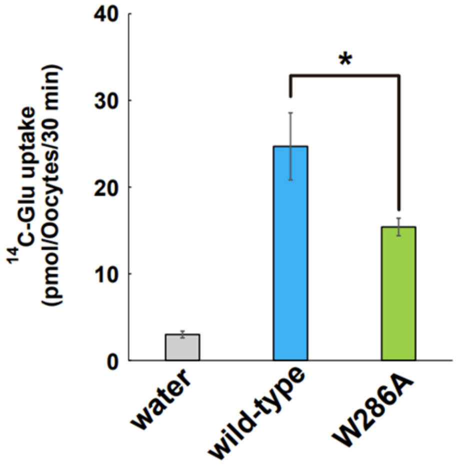

Cover Fig. 5 Glutamate uptake by wild type EAAT2 and the W286A mutant

Glutamate uptake by wild type EAAT2 and the W286A mutant, using *Xenopus* oocytes. W286A significantly decreases the uptake (*$p < 0.05$).

3. While the functional data for mutations in the inhibitor binding site is appreciated, Full dose response curves with a minimum of 7 concentrations of inhibitor should be used to properly delineate the effect of the mutagenesis on inhibition by WAY-213613. In line with this, saying that "inhibition by WAY-213613 was more or less affected by all three mutants" is too vague. Further data and interpretation are required.

Thank you for this comment. We agree with the Reviewer's comment regarding the importance of revealing the full dose response to discuss the detailed effects of the mutation more. However, there is a technical limitation in oocyte experiments. Because the results of amino acid

uptake experiment often show fluctuations from batch to batch, we must collect all of the data sets for the wild type and mutants using a single oocyte batch. However, the number of oocytes derived from one *Xenopus* is limited, and some oocytes after the cRNA injection cannot be used for uptake experiments due to their poor condition. Due to this technical limitation, we adopted four concentration points (0, 0.03, 0.1 and 0.3 micro-M) for oocytes expressing a construct (WT, I464V, L467I or V468I) and measured the transport activities (n=4–10 technical replicates for each concentration point) of each construct. In this assay, we used a total of 117 oocytes from one *Xenopus* and showed the result in Fig. 5b. Note that the expression and localization of each construct must also be investigated in the same batch, using a similar number of oocytes. To collect data for 7 inhibitor concentrations all of the constructs, we would need approximately twice as many oocytes, and this is actually beyond the technically possible amount using a single batch.

Even though the current data do not allow us to quantitatively discuss the influence of mutations on the sensitivity to WAY213613, we believe that the data are at least clear enough to show the drastic change in the L467I mutant and to demonstrate the critically important role of this residue for the interaction with WAY-213613. Related to this point, the sentence "inhibition by WAY213613 was more or less affected by all three mutants" in the original manuscript was modified to more accurately interpret the results in Fig. 5b.

MINOR COMMENTS

• Lines 52-53: "although excessive glutamate at synaptic clefts is associated with greater calcium influx." Is a confusing end to this sentence – please edit for clarity.

We have simplified this sentence.

• Lines 56-57: "Especially, EAAT2 …. is highly expressed at the plasma membrane of glial cells" should be replace with "EAAT2 …. is particularly highly expressed at the plasma membrane of glial cells"

We have corrected it.

• Lines 182-183: "a similar arrangement of the substrate-binding site could be observed in the outward-facing state of HsEAAT2." Saying "could be" implies there is data for this which there is not. Please replace with "may be" or "is likely to be".

We have corrected it.

Reviewer #3 (Remarks to the Author):

The manuscript by Kato et al. describes cryo-EM atomic models of human excitatory neurotransmitter transporter EAAT2 (SLC1A2) in the absence of substrate, as well as in the presence of a -commercially available- competitive inhibitor, WAY213613. A few functional experiments, with transporters heterologously expressed in oocytes, accompany the structural results.
Overall, the manuscript suffers from lack of novelty and insights on transport and/or pharmacological mechanisms of excitatory amino acid transporters. The work appears technically correct, but neither structural nor functional experiments are particularly challenging.

Answer:

We appreciate your comments on our manuscript. In this revised version, we updated three results. Firstly, we re-processes the data and successfully improved the resolutions of the substrate-free state and IFS-WAY213613 complex structures to 3.2 Å and 2.8 Å, respectively, which clarified the GDN and cholesterol binding. Next, our mutational analysis revealed, for the first tme, that the tryptophan residue coordinating the cholesterol molecule is important for the transport activity of EAAT2. Lastly, we showed that the WAY213613 inhibitor sensitivities for EAAT1 and EAAT3 are swapped by their mutations, confirming our hypothesis of the selective inhibition mechanism. Our point-by-point responses to your comments are below.

Major concerns:
1-There have been already several reports of experimentally-determined structures of human members of the SLC1A family (EAAT1, EAAT3, and ASCT2), and although the manuscript by Kato et al. reports the first structures of EAAT2, they lack new insights on the transport mechanism of the family. Inward-facing EAAT2 structure in the absence substrate (Fig. 1 and 2) nearly resembles the recently reported structure of EAAT3 (Qui et al., Sci Adv), but it is of lower resolution (~3.5 compared to 2.9 to angstroms). As the authors show in the manuscript, there are no "surprises" in the EAAT2 structure compared to published structural work on SLC1A proteins.

Thank you for the comment. Transport mechanisms of SLC1A, such as H^+-coupling and the one gate elevator mechanisms, have indeed been reported in previous papers, and based on them we have elucidated the structural basis for the inhibitory mechanisms by WAY213613, for the first time. We clarified that mechanism as well as other topics, such as how the known mutations cause diseases and the newly found conserved cholesterol binding sites (described below). Our research focuses on the molecular features of EAAT2 and the inhibitory mechanisms of WAY213613, and our results can contribute to EAAT2-related studies, including basic research. Furthermore, we re-performed the data processing of the IFS-WAY213613 state, based on the Reviewer #1's comments, and successfully improved the resolution to 2.8 Å.

2-The authors assign extra cryo-EM density to GDN and cholesterol molecules (Supp. Fig. 5).

However, the density blobs shown in that fig. do not appear to have molecular features to unambiguously assign those molecules. Furthermore, there is no functional-experimental effort whatsoever to test effects of lipids, in particular cholesterol, and the authors rely on published work by others to proposed vague modulatory roles of lipids on transport function.

Thank you for the comment. We more precisely assigned the cholesterol molecule and the head moiety of GDN, based on the shape and size of the improved densities, since the head moiety of cholesterol is different from that of GDN (Cover Fig. 1). During the review of our manuscript, the first cryo-EM structure of EAAT1 was published, in which similarly conserved tryptophan residue on TM5 coordinates cholesterol molecules (Cover Fig. 2), although the authors did not clearly mention them. Another structure of EAAT3 (PDB ID: 6S3Q) was released in PDB, but has not been published. Therefore, we could not perform a detailed comparison between EAAT2 and EAAT3. In this EAAT3 model, the cysteine residue corresponding to Trp286 of human EAAT2 coordinates the phosphocholine molecule (Cover Fig. 2). Considering our structures and other structural information about EAAT3 and EAAT1, tryptophan residues are important for the recognition of cholesterol molecules.

Reviewer 2 also requested a functional experiment of the cholesterol-coordinating residue in the revised version. Thus, we made a point mutant of Trp286 (W286A) and measured the expression level, localization and transport activities, using *Xenopus* oocytes. While the expression level and localization of W286A are similar to those of the wild type (Cover Fig. 4), the transport activity was markedly reduced (Cover Fig. 5). This result is supported by the previous report that membranes containing low levels of cholesterol have negative effects on the transport activity of EAAT2 (*Butchbach et al.*, 2004 and *Raunser et al.*, 2006). Our experimental results are presented in Supplemental Fig.5, and we have added the following from line 177.

"Similar cholesterol-binding sites were observed in ASCT2 and EAAT1 (refs. 27, 28, 32). Trp272 (the corresponding residue of EAAT2 is Met283, which is 1-turn apart from Trp286 of EAAT2) and Trp287 are located on the TM5s of ASCT2 and EAAT1, respectively, and these residues form similar interactions with the cholesterol molecules. The tryptophan residues located at the corresponding position of TM5 are highly conserved among eukaryotic SLC1A transporters (Supplemental Fig. 2), suggesting their importance among the family members. To assess its role, we mutated Trp286 to alanine and investigated the transport activity, using *Xenopus* oocytes. While both the expression level and localization of W286A were similar to those of the wild type, the glutamate uptake by the mutant was reduced (Supplemental Fig. 5**e-g**). This result suggests that the mutation of Trp286 has negative effects on the transport activity in plasma membrane environments."

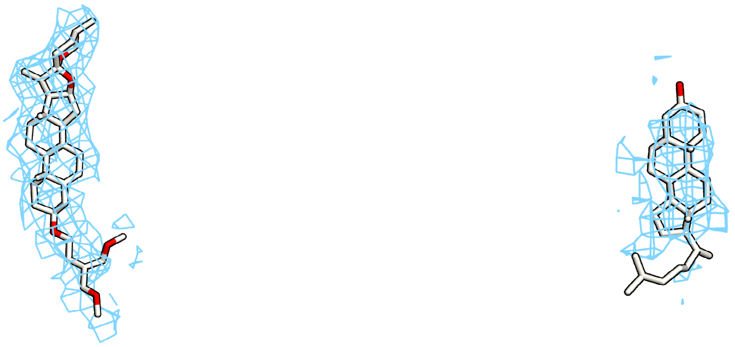

Cover Fig. 1 Density shapes and sizes of GDN and cholesterol

Densities of GDN (left) and cholesterol (right). GDN and cholesterol consist of six and four aromatic rings, respectively, and the shape and size of each density correspond to each molecule.

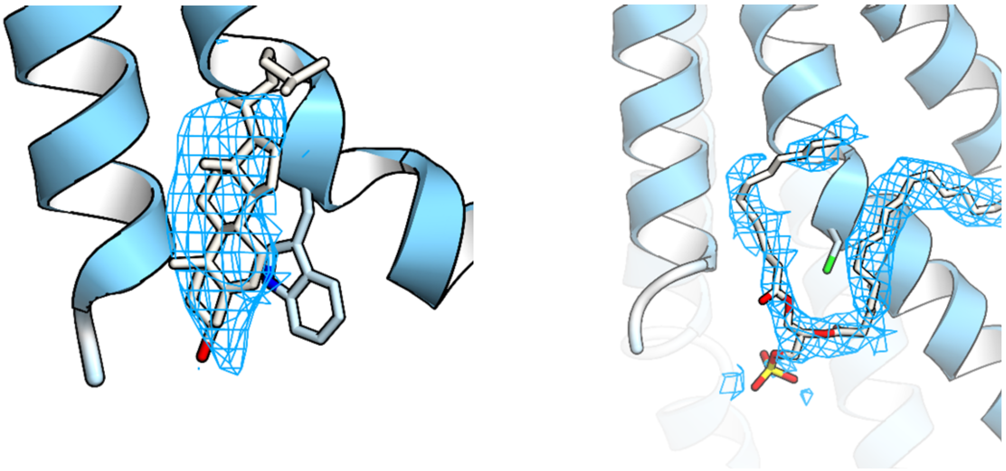

Cover Fig. 2 Cholesterol and lipid interactions with TM5 of EAAT1 and EAAT3

Densities of cholesterol (left) and phospholipid (right) near TM5s of EAAT1 and EAAT3.

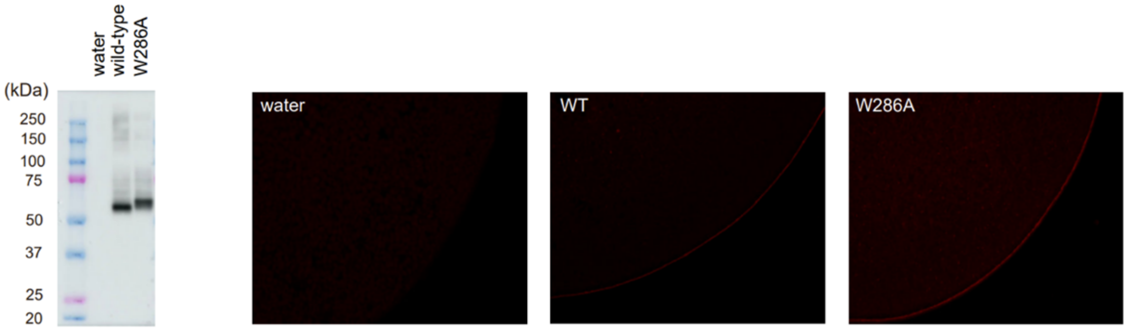

Cover Fig. 4 Expression levels and localizations of the wild type and the W286A mutant of EAAT2

Western blotting (left) and immunofluorescence analyses (right) of wild type EAAT2 and the W286A mutant. Both the expression level and localization of the wild type are similar to those of W286A.

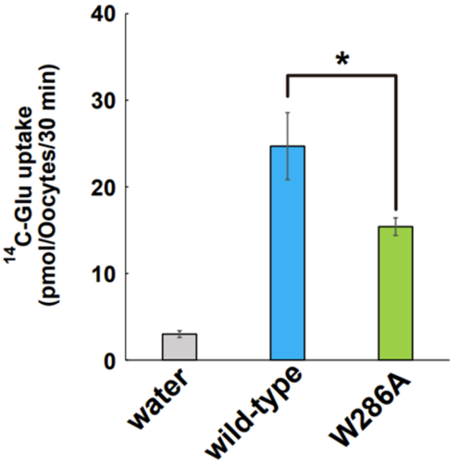

Cover Fig. 5 Glutamate uptake by wild type EAAT2 and the W286A mutant

Glutamate uptake by wild type EAAT2 and the W286A mutant, using *Xenopus* oocytes. W286A significantly decreases the uptake (*$p < 0.05$).

3-The molecular mechanism underlying competitive inhibition in this family of transporters, whereby the inhibitor precludes movements of HP2, was nicely demonstrated by the Gouaux laboratory back in 2007 (Boudker et al., Nature 2007), and it has been later confirmed in several reports. WAY213613 was developed more >15 years ago, and was then shown to be a competitive glutamate-transport inhibitor using functional assays (Dunlop et al., Mol Pharm, 2005). Hence, the proposed WAY213613 inhibitory mechanism in the manuscript (Fig. 4 and Fig. 6) is just confirmatory.

Thank you for the comment. In the previous report by Boudker *et al.*, the Benzyloxy group

of the TBOA inhibitor pushes the HP2 loop away from the substrate binding site to prevent its closure. Similarly, the non-amino acid group of TFB-TBOA also pushes the HP2 loop to interfere with its closure [*Canul-Tec et al., Nature 2017* and unpublished structure (PDB ID 6S3Q) of EAAT3 with this inhibitor]. While WAY213613 also interferes with the HP2 loop closure, the underlying inhibitory mechanism is different from those of TBOA and TFB-TBOA. In the previous TBOA- and TFB-TBOA-bound structures, the HP2b helix collapses the cavity near the amino acid binding site. However, in the WAY213613-bound EAAT2 structure, the movement of the HP2b helix is inhibited by the non-amino acid group (the BDP moiety) of WAY213613 in the cavity, resulting in the indirect interference of the loop closure. We have added this explanation in line 262–275 (the following text). Additionally, although WAY213613 was developed over 15 years ago (published in 2005), the selective mechanism has never been clarified until our present work. In the present paper, we have elucidated the structural basis for the selective inhibitory mechanism of WAY213613 for the first time. Therefore, we have added the following description in the revised text from line 262.

"The comparison among this IFS-WAY213613 state of EAAT2 and the Asp-bound states of EAAT1 and EAAT3 revealed that the end of HP2b slightly moves towards TM7 and TM8 upon aspartate binding, which accompanies the closing of the HP2 loop gate (Supplemental Fig. 10**b, c**). The amino acid moieties of other competitive inhibitors, TBOA and TFB-TBOA, are also captured by the glutamate-binding sites of the transport domains[19,26]. In the TFB-TBOA-bound state of EAAT1, the non-amino acid moiety of TFB-TBOA inhibits the movement of the HP2 loop. However, the arrangement of HP2b could not accommodate the BDP moiety (Supplemental Fig. 10**d**). While WAY213613 is the selective inhibitor for EAAT2, TBOA and TFB-TBOA broadly interfere with SLC1A transporters[54,55]. Considering our results from EAAT1–3, the selective effects on WAY213613 are related to the environments of their cavities (Fig. 5**b, c**), suggesting that the inhibitory mechanisms of WAY213613 are different from those of other non-selective inhibitors, such as TBOA and TFB-TBOA. When the BDP moiety is captured by the cavities, the moiety is likely to sterically interfere with the movements of the HP2b helix, indicating that WAY213613 indirectly prevents the HP2 loop gating of EAATs."

4-The blob of density corresponding to bound WAY213613 (Fig. 4) seems to lack molecular features to reveal the exact atomic coordination of this molecule, and a more thorough mutagenesis study is necessary to uncover the molecular determinants underlying the ~ 50-fold selectivity of this compound for EAAT2 over EAAT1 and 3 (Dunlop et al., Mol Pharm, 2005). Importantly, in my view, the potential therapeutic use of WAY213613 for cancer therapy in peripheral organs, as suggested in the manuscript, is overshadowed by the risk of a central neurotoxic effect on excitatory synapses. The question remains, if it is really relevant to understand the molecular determinants of WAY213613 selectivity for EAAT2.

In conclusion, the manuscript lacks important mechanistic functional or pharmacological insights on EAATs, and I would not recommend publication.

Thank you for the comment. First, we have re-processed and successfully improved the resolution of the WAY213613 complex, resulting in a clearer density for this inhibitor (Cover Fig. 7). Regarding the excitotoxicity risk of WAY213613, Reviewer 2 also asked a similar question. As this inhibitor has not been examined in animal experiments, we predicted whether WAY213613 permeates the blood brain barrier (BBB) by using the BOILED-Egg program, which shows the BBB permeability potential of compounds. Based on the results, WAY213613 would not permeate the BBB (Cover Fig. 3). Because this is just a prediction, it should be carefully validated in future studies to pursue the possibility of clinical applications of WAY21361. This information could prompt further studies to develop WAY213613 (and possibly its derivative compounds) as anti-cancer drugs. Therefore, we have included the following description and result in line 310–329 (the following text).

“Indeed, the different residues around the tip of the BDP are related to the WAY213613 sensitivities (Fig. 5), and these results could support strategies for BDP modifications. WAY213613 has not yet been tested in animal experiments to evaluate its potential to permeate the blood brain barrier (BBB) and cause excitotoxicity. The BBB permeability of EAAT2 inhibitors would have the potential risk of excitotoxicity. However, WAY213613 is predicted to not pass through the BBB, based on the SwissADME server, which includes the BOILED-Egg prediction program judging BBB permeability based on the lipophilicity and polarity of small molecules[58,59]. The relationship between cancers and glutamate is gradually being elucidated, highlighting the possibility of anti-cancer drug development using glutamate-targeted but BBB-impermeable compounds[60]. Recent studies have revealed that EAAT2 is upregulated in at least three types of tumors (gastric, colorectal and breast cancers)[37–40], and other cancers might utilize EAAT2 and/or other EAATs for their aggressive phenotypes including, but not limited to, malignant progression and resistance to chemo- and endocrine therapies. Consistently, research published in 2022 reported that EAAT3 enhances tumor angiogenesis[61]. Therefore, although further experiments to reveal the BBB impermeability and anti-cancer effects are required, WAY213613 and its derivative compounds may pave the way for future research and/or strategies for cancer therapies. Furthermore, since the recent work suggested that EAAT2 is potentially expressed in retinal and peripheral tissues (liver, lung, and testis)[62], such selective inhibitors will reveal more details of the physiological roles of EAAT2 in the extracellular glutamate homeostasis of not only the brain but also other organs.”

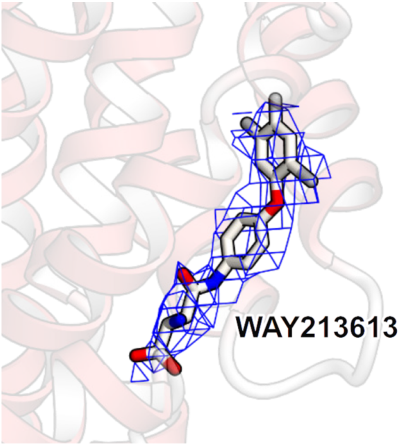

Cover Fig. 7 Density of WAY213613

Raw density of WAY213613 in the new map.

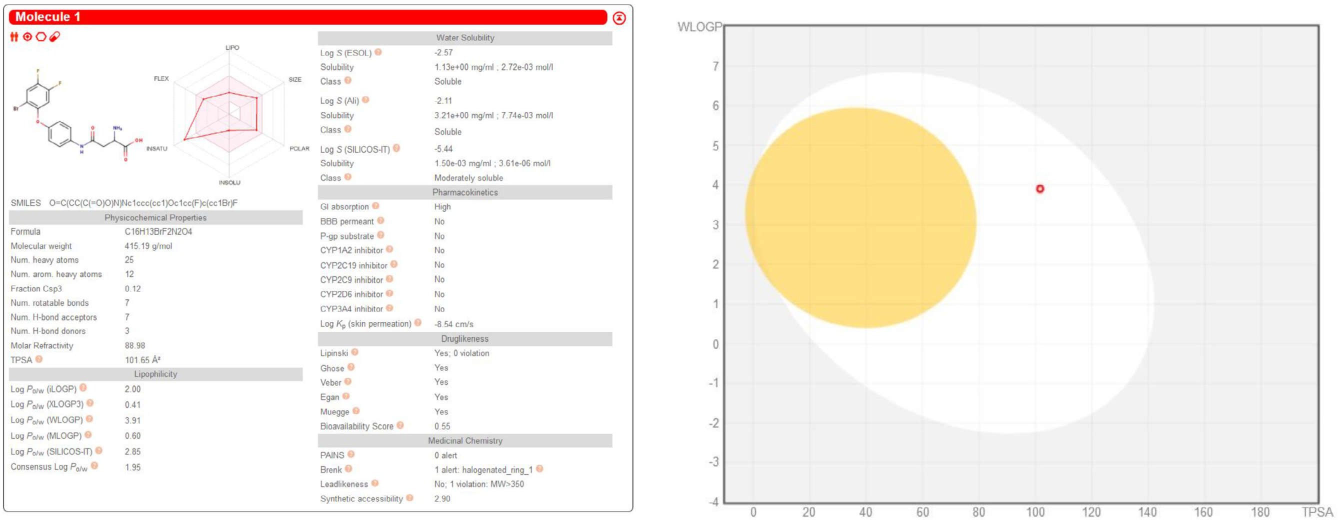

Cover Fig. 3 BBB impermeability of WAY213613

SwissADME results (left). In the Pharmacokinetics session, although "GI (gastrointestinal) absorption" is high, "BBB permeant" shows "No", indicating the BBB impermeability of WAY213613. BOILED-Egg program results (right). This chart demonstrates two values, tPSA (horizontal value: topological polar surface area) and WLOGP (vertical value: log P method developed by Wild- man and Crippen). White and yellow areas correspond to gastrointestinal absorption and BBB permeability, respectively, and the red dot corresponds to WAY213613.

To address your comment about the selectivity, we investigated the WAY213613-sensitivities of EAAT1 and EAAT3, using their wild types and mutants with isoleucine residues (their positions correspond to Leu467 of EAAT2) changed to leucine residues. Although two concentrations

of WAY213613 (1.5 and 15 μM) did not significantly change the glutamate uptake by EAAT1, the I468L mutant showed much higher sensitivity. Wild type EAAT3 showed slight inhibition by WAY213613, but the inhibitory effect was much more prominent in the I436L mutant (Cover Fig 8). These results confirm our hypothesis that the leucine residue is essential for the WAY213613 selectivity. We have added this result and the following description in lines 232–244 (the following text) and Fig. 5.

“According to the sequence alignment, the residue corresponding to Leu467 in EAAT2 is substituted with isoleucine in EAAT1, EAAT2 and all other SLC1A transporters. Since the L467I mutation of EAAT2 decreased the WAY213613 sensitivity, the “EAAT2-like” mutations (isoleucine to leucine) of EAAT1 and EAAT3 may change the sensitivities to WAY213613. To assess this possibility, we generated “EAAT2-like” mutants of EAAT1 (I468L) and EAAT3 (I436L) and measured their glutamate uptake in the presence of WAY213613, using oocytes. While the uptake activities of wild type EAAT1 at 1.5 and 15 μM were not significantly affected, those of the I468L mutant were decreased in a concentration-dependent manner (Fig. 5c and Supplemental Fig. 6c). In addition, whereas the wild type EAAT3 showed only a slight decrease in the uptake activities by WAY213613, the decrease became much more prominent in the I436L mutant. Therefore, these leucine mutations in EAAT1 and EAAT3 remarkably change the WAY213613 sensitivities. These results suggest that the residues forming the cavities are closely related to the sensitivities of the EAAT subtypes to WAY213613.”

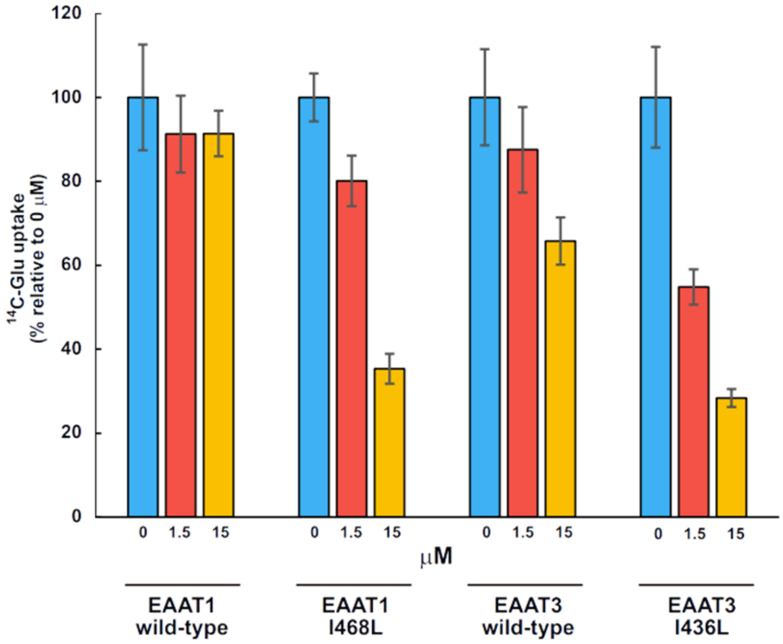

Cover Fig. 8 Sensitivities of wild types and mutants of EAAT1 and EAAT3

Glutamate-uptake assay for point mutants of EAAT1 and EAAT3 treated with WAY213613. Values of the vertical and horizontal axes are relative to each 0 μM and the concentration points (0, 1.5 and 15 μM) of WAY213613, respectively.

REVIEWERS' COMMENTS

Reviewer #1 (Remarks to the Author):

The authors have addressed my concerns and I have no more comments.

Reviewer #2 (Remarks to the Author):

I thank the authors for their comments.

While overall they have answered my queries, I somewhat disagree with the authors reply regarding the full dose response curves. Yes, expression in oocytes does vary week to week, but this can easily be normalized – i.e., normalize all uptake values to the uptake of 100 uM substrate in WT for each given week. In fact, the fact that all of these experiments have been done in one setting raises the issue that biological replicates have not been performed – these experiments should be conducted over at least 3 different batches of oocytes. For the sake of a better, more scientifically rigorous manuscript, I would suggest repeating the experiment with full dose response curve and utilizing the normalization described above to negate any differences between oocyte batches.

Reviewer #3 (Remarks to the Author):

The authors have made an honest and significant effort to improve the revised manuscript. In particular, by re-processing the cryo-EM data, they have been able to substantially improve the overall resolution of the maps, facilitating the interpretation of non-protein density assigned to lipid and detergent molecules. Further mutagenesis and functional experiments now support a regulatory role of cholesterol on transmitter transport. Finally, the authors predict that the EAAT2-selective inhibitor WAY213613 would poorly cross the BBB, leaving some hope for a therapeutic use of this small-compound molecule in cancer therapy.

I don’t have further concerns on the manuscript.

Responses to Referees

Reviewer #1

The authors have addressed my concerns and I have no more comments.

Answer:

Thank you very much for your kind review on our revised manuscript.

Reviewer #2

I thank the authors for their comments.

While overall they have answered my queries, I somewhat disagree with the authors reply regarding the full dose response curves. Yes, expression in oocytes does vary week to week, but this can easily be normalized – i.e., normalize all uptake values to the uptake of 100 uM substrate in WT for each given week. In fact, the fact that all of these experiments have been done in one setting raises the issue that biological replicates have not been performed – these experiments should be conducted over at least 3 different batches of oocytes. For the sake of a better, more scientifically rigorous manuscript, I would suggest repeating the experiment with full dose response curve and utilizing the normalization described above to negate any differences between oocyte batches.

Answer:

Thank you for the valuable comment. For the functional analyses of transporters and channels, using a single batch of oocytes has been adopted as a practical and conventional method because oocytes have different biological backgrounds from batch to batch. Accordingly, as Reviewer#2 pointed out, careful checking of the reproducibility using multiple batches of oocytes will be critically important. In the previous submission, however, the sensitivity of EAAT2 WT and mutants for WAT213613 in Fig. 5B was compared only with two batches of oocytes. We appreciated the comment and repeated the experiment one more time to verify that the results are well reproducible in all the three independent experiments.

We agree with the comment of Reivewer#2 that the comparison using full dose-response curves would be ideal, if technically feasible. However, as we already mentioned in the previous "Response to Referees Letter", there is a limitation in the number of oocytes available in a single batch. In this regard, the normalization method suggested by Reviewer#2 would be a certainly effective way to circumvent this technical issue. Still, we cannot either manipulate or assess the intracellular

conditions that may affect the function of EAATs, such as the endogenous glutamate and aspartate and the coupled ions (H^+, Na^+, and K^+) in oocytes. The difference in the background uptake of glutamate in water-injected oocytes between batches also suggests the considerably distinct conditions of oocytes, possibly such as the expression of endogenous glutamate transporters, the integrity of the plasma membrane against solutes, and so on. Moreover, in our experience, different transport activities between batches cannot be exclusively attributed to the distinct expression levels of the transporter. Carefulness is required for the normalization between batches of oocytes because there seem to be contributions of such uncertain factors, most likely reflecting the different physiological conditions of frogs and/or oocytes.

Reviewer #3 (Remarks to the Author):

The authors have made an honest and significant effort to improve the revised manuscript. In particular, by re-processing the cryo-EM data, they have been able to substantially improve the overall resolution of the maps, facilitating the interpretation of non-protein density assigned to lipid and detergent molecules. Further mutagenesis and functional experiments now support a regulatory role of cholesterol on transmitter transport. Finally, the authors predict that the EAAT2-selective inhibitor WAY213613 would poorly cross the BBB, leaving some hope for a therapeutic use of this small-compound molecule in cancer therapy.

I don’t have further concerns on the manuscript.

Answer:

Thank you for the kind review on our revised manuscript.
